# The potential of thermally expanded graphite in oil sorption applications

**DOI:** 10.1039/d4ra00049h

**Published:** 2024-05-21

**Authors:** Moammar Elbidi, Mohamad Amran Mohd Salleh, Suraya Abdul Rashid, Mohamed Faiz Mukhtar Gunam Resul

**Affiliations:** a Sustainable Process Engineering Research Centre (SPERC), Department of Chemical and Environmental Engineering, Faculty of Engineering, Universiti Putra Malaysia 43400 UPM Serdang Selangor Malaysia elbidim@gmail.com; b Institute of Nanoscience and Nanotechnology (ION2), Universiti Putra Malaysia 43400 UPM Serdang Selangor Malaysia asalleh@upm.edu.my

## Abstract

An oil spill occurs when liquid petroleum hydrocarbons are released into the environment, whether accidentally or intentionally, in substantial quantities. The impact of an oil spill on the ecosystem is significant and should not be underestimated. Various techniques are employed to address oil spills, including mechanical, physical, biological, and physicochemical methods. Among these techniques, adsorption is considered the most suitable approach. Adsorption is promising due to its simplicity, ease of use, high removal capacity, and rapid pollutant removal. An excellent adsorbent material exhibits unique characteristics that enhance its efficacy in liquid adsorption. Sorbents are categorized into synthetic and natural types. Porous carbon materials, especially expanded graphite, are widely utilized in wastewater treatment due to their micropores and exceptional adsorption capacity. The distinctive properties of expanded graphite, including its low density, high porosity, and electrical conductivity, have garnered significant global attention for various potential applications. In essence, expanded graphite offers a powerful and practical approach to oil spill cleanup due to its efficient oil adsorption, selective targeting, ease of use, and potential reusability. This review article summarizes the preparation techniques, structure, and properties of expanded graphite. It also delves into recent advancements in using expanded graphite for oil spill cleanup. The article concludes by outlining potential future directions in this field and discussing the commercial viability of some of these techniques.

## Abstract 2

Porous carbon materials, especially expanded graphite, are widely utilized in wastewater treatment due to their micropores and exceptional adsorption capacity.

## Graphical abstract

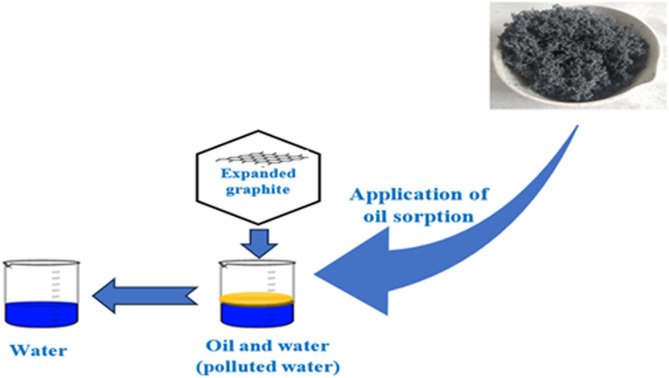

## 1. Introduction

Global energy consumption is enormous and gradually increasing annually, reflecting the immense amount of power we use to fuel our needs. Fossil fuels like coal, oil, and natural gas still dominate the energy mix, despite the steady rise of renewable sources. This dependence on fossil fuels, driven by our ever-growing energy demands, increases the risk of oil spills during transportation and extraction, posing a significant threat to our environment.^1–4^

An oil spill occurs when liquid petroleum hydrocarbons are released into the environment, either accidentally or intentionally, in significant amounts. Such spills can take place in various locations, including marine environments such as oceans, seas, or rivers, as well as on land from pipelines or storage sites. The impact of oil spills on ecosystems, wildlife, human well-being, and the economy makes them a major environmental issue.^5,6^ Oil spills have harmful effects not only in marine environments but also in freshwater ecosystems, such as lakes, rivers, and wetlands. The term “aquatic” encompasses both marine and freshwater environments.^7^ When an oil spill occurs in an aquatic environment, it poses a threat to organisms residing on or near the water surface, as well as those living underwater. Additionally, the spill can disrupt the food chain, impacting valuable resources for human consumption. The severity of the spill's impact is influenced by several factors, including the characteristics of the oil itself and natural conditions such as water temperature and weather. Different types of habitats exhibit varying sensitivities to oil spills.^1,5^

Oil spills pose severe environmental catastrophes, resulting in substantial and long-term consequences for the affected ecosystems, socio-economic activities, and the overall environment.^2,8^ The long-term impact of environmental pollution emphasizes the urgency to enhance measures for the protection of coastlines and marine ecosystems. Various materials have been employed in endeavours to restore marine and wildlife habitats, although with varying degrees of success, in the context of oil spill cleanup and recovery.^8–10^

### 1.1 Oil spills remediation

Various techniques are used to tackle the oil spills which include mechanical, physical, biological, and physic-chemical methods. The mechanical technique involves the use of specialized mechanical devices, known as “skimmers,” that have been effectively designed and deployed on floating platforms to collect substantial oil layers from the water's surface. However, the collection of thin oil layers, particularly during turbulent weather conditions, and even from soil surfaces, requires the use of sorbents.^10^ In addition, skimmers are complex in application, require proper maintenance and may be jammed and clogged with water. In the application area, skimmers considered rapid and effective are used on boats to remove the oil from the water's surface once the booms have confined it.^11^ To summarize, mechanical techniques are most effective in calm water conditions where there is minimal wind or current, such as in coastal waters, estuaries, and port basins. It is important to note that the design of the booms also significantly influences their performance and efficiency.^12^

Chemical treatment is employed to diminish the efficacy of blocking sunlight and air from the impacted surfaces by dispersing oil slicks. Chemical coagulation and flocculation have good removal efficiency, suitable to all weather situations and applicable for different types of oils; however, they are associated with high initial and operating costs and are labour-intensive, leading to significant energy consumption. Additionally, they can generate secondary pollutants.^13,14^ The chemical treatment technique is considered less reliable for practical application.^11^

Bioremediation for oil spills is a technique in which bacteria and fungi break down complex compounds into smaller ones.^15^ Biological treatment exhibits high oil removal efficiency and low operating costs. However, it requires precise control of temperature and pH, and it necessitates extended treatment time, skilled operators, and a large space.^13^ In terms of application, this method is typically not used for spills in deep seas and is gradually implemented as the oil approaches the shoreline.^11^ Abiotic environmental factors, such as inadequate nutrient levels including phosphate and fixed forms of nitrogen, extremely low temperatures, and insufficient oxygen, impose limitations on the effectiveness of bioremediation.^12^

### 1.2 Oil sorption technique

According to the reviewed literature, conventional treatment methods exhibit poor performance and have other significant drawbacks, including lengthy treatment times, extensive land requirements, and high capital costs. Even though these techniques are employed for oil spill remediation, adsorption is considered the most suitable method.^16^ The process of oil sorption using adsorbents is a practical method for addressing oil slicks in areas affected by spills. Adsorption has been viewed as a promising technique due to its simplicity, ease of use, high removal capacity, and rapid removal of pollutants.^16,17^ Generally, the process of oil adsorption in sorbents consists of three chemical stages. The first stage is diffusion, where oil molecules penetrate the surface of the sorbent. The second stage involves the trapping of oil within the structure of the sorbent through capillary action. In the final stage, oil droplets accumulate within the porous and rough structures of the sorbent.^13^

An excellent adsorbent material possesses unique characteristics that make it highly effective in absorbing liquids. These characteristics include a high absorption capacity, which allows it to absorb and retain a significant amount of oil relative to its own weight. It also has a fast absorption rate, quickly drawing in the oil upon contact, minimizing the spread of the spilled substance and reducing further contamination. Additionally, it has good retention properties, ensuring that the absorbed liquid is contained within the material without significant leakage or release.^12,13^ Furthermore, an excellent adsorbent material is environmentally friendly and biodegradable. It does not introduce additional harm to the environment and naturally degrades over time. Cost-effectiveness is another important factor, as the material should provide a balance between performance and affordability. It should offer a reasonable cost per unit of absorbency, making it a practical choice for spill management. Lastly, recyclability is a desirable characteristic, as it promotes sustainability and reduces waste.^18–20^ By meeting these specifications, an adsorbent material can be considered excellent in terms of its effectiveness, efficiency, and suitability for managing spills across various industries and applications.

### 1.3 Adsorbents used to treat oil spills

Shokry *et al.* (2020); prepared and tested the absorption of nano-magnetic activated carbon hybrid material, and the result demonstrated of oil adsorption capacity of 30.2 g g^−1^.^21^ Soliman *et al.* (2020) conducted a study on the absorption capacity of wood sawdust-coated magnetite nanoparticles that were functionalized with stearic acid. In their research, they determined that the maximum absorption capacity reached 41.22 g g^−1^ of crude oil^16^.^15^ modified of raw flax fibre through acetylation and microwave energy enhances its hydrophobic properties, resulting in improved oil sorption capacity (24.54 g g^−1^) compared to raw (13.75 g g^−1^) and microwave fibre (17.42 g g^−1^). Table 1 illustrates the oil absorption capacity of different sorbents.

**Table 1:** Comparison of adsorption capacity of various adsorbent materials

Grade	Sorbent	Sorption capacity (g g^−1^)	Oil type	Ref.
High capacity, >50 g g^−1^	Carbon fibre aerogel from bamboo	70–85	Diesel–crude	22
	Graphene sponge	68.5	Lubricating	23
	Exfoliated graphite by microwave irradiation	56	Engine	24
	Functional graphite sheets	47–63	Diesel – engine	25
Med-capacity 25–50 g g^−1^	TiO_2_-coated nanocellulose	40	Organic solvents	26
	Polyimide/graphene aerogel	37.44	Motor	27
	Nanofibril aerogels	24–46	Heavy	28
	Magnetic cellulose aerogel/TiO_2_ aerogel	28	Diesel (paraffin)	29
	Magnetic graphene/CNT foam	27	Motor	30
Low capacity, <50 g g^−1^	Polysulfone/NiFe_2_O_4_ nanostructured fibrous	15.11	Motor	31
	Expanded perlite	3.5	Light crude	12
	Magnetic cobalt ferrite nanoparticles	3.6	Used motor	20
	Palm fatty acid functionalized Fe_3_O_4_ nanoparticles	3.5	Lubricant	18

Sorbents can be divided into synthetic and natural types. Currently, in developed countries, the most effective sorbents are made of foamed synthetic materials. Various materials have been proposed as adsorption agents, including nanomaterials, nanocomposites, nanoparticles, clays, biopolymers, metal–organic frameworks, and zeolites. Among carbon-based materials, porous carbon materials are commonly used in wastewater treatment due to their micropores and excellent adsorption capacity. Activated carbon, in particular, has been recommended as an ideal agent for removing contaminants from water. However, its production and regeneration can be quite expensive.^9^ Similarly, special molecules such as carbon nanotubes and graphene have demonstrated exceptional performance, but their current cost renders them impractical for utilization in oil spill remediation.^12^

The intrinsic properties of expanded graphite, including its low density, high porosity, and electrical conductivity, have received significant global attention for their potential applications. This material shows promise in various fields, including fuel cells, electromagnetic interference shielding, catalysts, vibration damping, supercapacitors, biomedical materials, and as a spilled oil adsorbent.^10,32^

## 2. Expanded graphite

It is important to acknowledge that conventional graphite typically has limited applications and exhibits limited efficiency in different applications. While the electrical and thermal conductivities properties can improve with higher concentrations of graphite, as well as the mechanical properties. To achieve enhanced efficiency, there is a need to expand the layered graphite flakes, which possess a higher aspect ratio and a larger specific surface area. Despite its versatility and wide range of applications, graphite still has limitations in certain areas. However, with further advancements, graphite has the potential to become an even more exceptional material. One such development is expanded graphite, which features a vermicular or worm-like structured non-toxic layered material. This material possesses excellent flexibility, high chemical tolerance, and exceptional thermal shock resistance.^33–36^

**Fig. 1:**
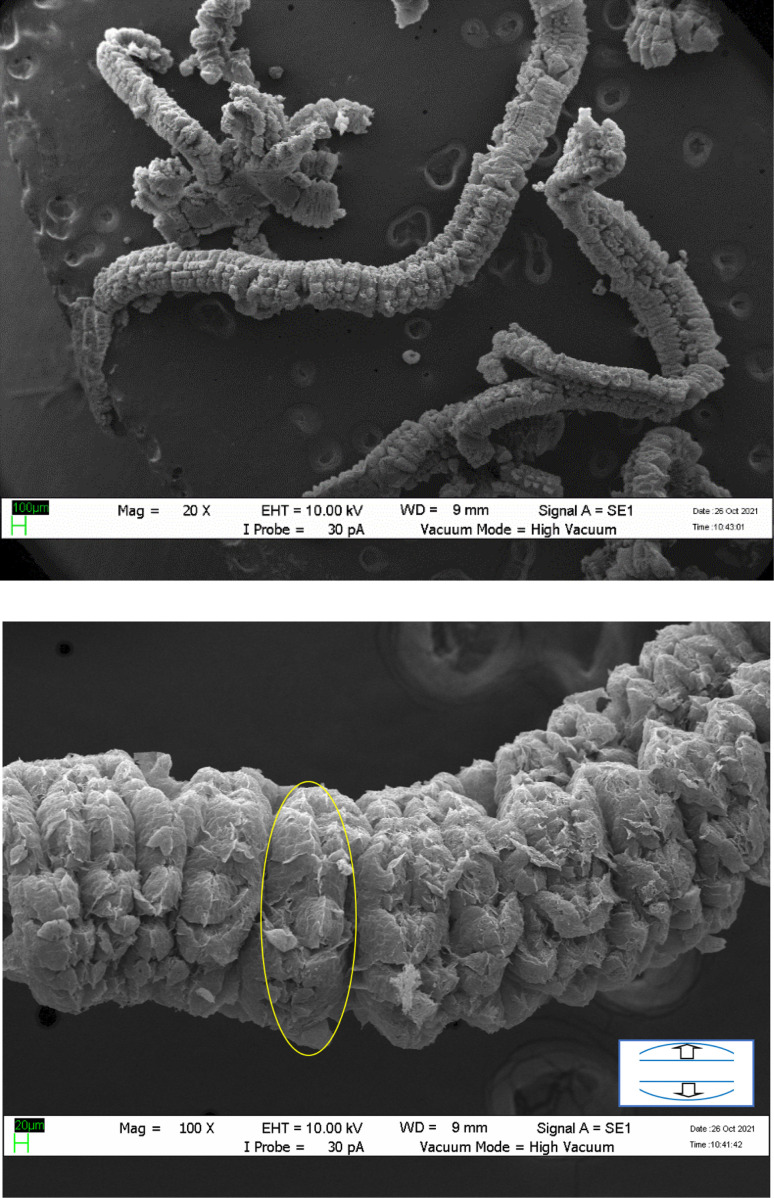
SEM photographs of expanded graphite. Reprinted with permission from ref. 75.

Expanded graphite is a form of graphite that has been treated with an intercalation agent, such as sulfuric acid as displayed in Fig. 1,^37–42^ nitric acid^24,36,43^ and perchloric acid^17,44–46^ or electrochemical methods of natural flake graphite.^47,48^ For successful graphite expansion, overcoming the van der Waals attractions that exist between adjacent layers is required.^34,49^ Expansion is often accomplished by applying additional external forces to overcome the attractive van der Waals interactions between layers. The commonly used techniques are ultrasonication and thermal treatment. During ultrasonication, shear forces and cavitation (the growth and collapse of micrometer-sized bubbles) act on the bulk material and induce expansion.^50,51^ In the thermal expansion of graphite, the pressure due to the decomposition of the functional groups and intercalates between layers overcomes the van der Waals attractions and results in expansion.^52–54^ There are a variety of successful expansion techniques that may be categorized into three major classes: mechanical, thermal, and electrochemical. The most effective technique to decrease these attractions is by expanding the distance between the adjacent layers *via* oxidation and chemical intercalation reactions^55–58^ which strong acid such as sulfuric acid, nitric acid, or perchloric acid used as the intercalation agents in combination with hydrogen peroxide, potassium permanganate, or nitic acid as the oxidation agents, followed by conventional or microwave heating.^59,60^

Expansion process results in a highly porous material with a unique set of properties that make it useful in a variety of industrial applications.^34,61^ EG is a very porous, worm-like, and light material with typical apparent densities of 0.002–0.01 g cm^−3^.^62–64^ This material “EG” with several existing and potential applications in energy storage,^59,65^ hydrogen storage,^66^ fuel cell,^67^ sensor,^68^ catalyst,^69^ biomedical materials,^70,71^ filler,^72^ and adsorbent.^73^ One of the most significant properties of expanded graphite is its ability to act as a flame retardant. The expanded structure of the graphite creates a barrier that can help prevent the spread of flames and limit the damage caused by fires. This makes it an ideal material for use in applications such as fireproofing insulation, gaskets, and seals.^74^

EG is also an excellent thermal conductor, which makes it useful in heat management applications. It can be used as a heat sink to dissipate heat away from electronic components and other heat-generating devices.^76,77^ Another important property of expanded graphite is its chemical stability. It is resistant to most chemicals and can withstand high temperatures without degrading. This makes it useful in a range of harsh environments, including those found in the chemical processing and oil and gas industries.^78^ Finally, expanded graphite is a versatile material that offers a unique combination of properties, including flame retardancy, thermal conductivity, and chemical stability. These properties make it useful in a range of industrial applications where high-performance materials are required.

EG can be obtained by conventionally chemical oxidation process using concentrated sulfuric acid and nitric acid as intercalating agent and oxidant agent respectively, with an expansion volume up to several hundred times.^73,79,80^ These conventional processes beside all the known drawbacks from emitting some unfriendly gases, complex processing, long reaction time, and high-energy consumption.^37,38,60^ Moreover, the strong intercalating agents might damage the structure of graphite and cause formation of some oxygen-containing functional groups on the surface of produced EG which impacts the end use of some applications of EG.^38,81^ Thus, custom-made EG materials with control of these functional groups can be prepared depending on the requirements of corresponding applications.^81^ In this regard, an EG fabrication technique with environmentally acceptable characteristics should be developed.^17^ Recently, preparation of EG by other acids was reported. Many researchers have proposed ways to reduce or even eliminate the sulfur content of EG. Most of these methods adopt nitric acid,^43^ phosphoric acid,^43,74^ perchloric acid,^45,60,82^ hydrochloric acid,^83^ boric acid,^51^ peroxyacetic acid,^84^ and so forth, which may introduce new pollutants or hazardous chemicals.

### 2.1 Graphite intercalation compounds “GICs”

Graphite can react with a variety of chemical substances to form different types of compounds. These compounds can be classified into three groups: surface compounds, substitutional compounds, and intercalation compounds.^33^ Surface compounds, these are formed when chemical substances react with the surface of the graphite. Surface compounds are usually formed by oxidation or reduction of the graphite surface. Examples of surface compounds include graphite oxide and reduced graphite oxide. Substitutional compounds, these are formed when atoms or molecules are substituted for carbon atoms in the graphite lattice. The resulting compounds have different properties from graphite, such as different electrical conductivity and hardness. Examples of substitutional compounds include silicon carbide (SiC) and boron carbide (B_4_C). Intercalation compounds, these are formed when chemical species are inserted between the graphite layers. Intercalation compounds have unique properties such as high electrical conductivity and can be used as battery anodes or in electronic devices. Examples of intercalation compounds include graphite fluoride and graphite intercalation compounds with alkali metals (*e.g.*, Li, Na, K).^33,34,49,85^

**Fig. 2:**
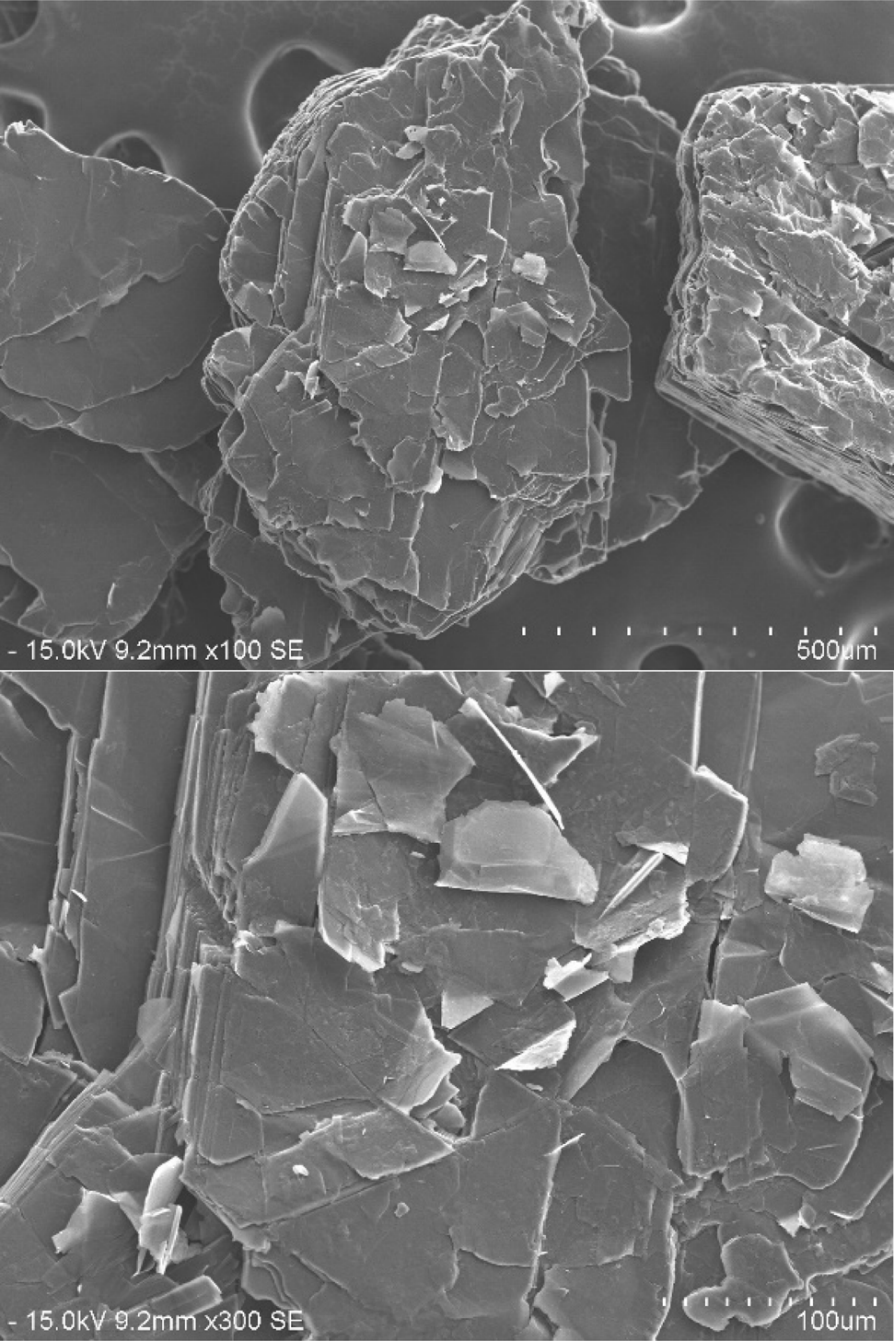
SEM photograph of GICs. Reprinted with permission from ref. 42.

Graphite intercalation compounds “GICs”, as exposed in Fig. 2, are created by inserting foreign species like atoms, ions, or molecules between the layers of the graphite lattice.^34^ These reactions are called intercalation.^49^ Intercalation compounds are produced by introducing a foreign substance into the host lattice. However, the structure of these compounds is different as the bond is a charge-transfer interaction.^86,87^ This electronic interaction results in a significant increase in electrical conductivity in the ab directions.^63,88^ In this structure, the intercalated species occupy the interplanar interstitial sites between the layers, while the layered structure of the graphite lattice remains mostly unchanged.^33,86^

The first reported synthesis of a graphite intercalation compound dates back to 1841 by Schaffautl.^86^ However, the first organized studies of these compounds began in the early 1930s with the introduction of X-ray diffraction techniques for stage index determinations (Hoffman and Frenzel 1931, Schleede and Wellman 1932). Although the systematic examination of their physical properties began in the late 1940s, it was not until recent years that research on graphite intercalation compounds has become a globally active field of study.^34^ Direct synthesis is typically employed to produce the simplest binary and ternary compounds, whereas a variety of intercalation techniques are used to prepare the more complex materials. Because of their extreme reactivity, most graphite intercalation compounds are manufactured and stored in ampoules pressurized with an inert gas, a vacuum, or intercalate vapor. The stability of samples is considerably improved by cooling them (for instance, to liquid nitrogen temperatures).^34^

Graphite intercalation compounds are classified into two types, namely the ionic compounds and the covalent compounds. The ionic compounds (*e.g.*, graphite intercalated with sulfuric acid, which is also known as graphite bisulfate) are characterized by charge transfer between the intercalate and the graphite so that a low degree of ionic bonding occurs.^34,61^ Moreover, many of the intercalates of this group retain their molecular identity in the graphite lattice, so that the nature of the ionic bonding is more complicated than that in many of the totally ionic solids, where simple ions are involved.^49^

#### 2.1.1 Staging

The staging phenomenon is the most significant and distinctive property of graphite intercalation compounds, which involves the periodic arrangement of intercalate layers between the graphite layers. The distance between adjacent intercalate layers is called the stage height, and the number of graphite layers between two adjacent intercalate layers is called the stage number.^34,63^

**Fig. 3:**
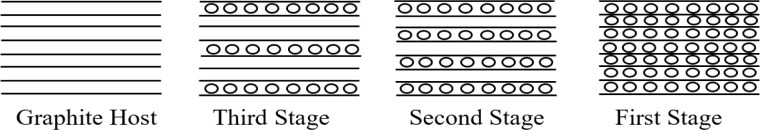
Schematic illustration of staging in intercalates compounds. The solid line represents the host layer, the dots a gust layer. Reprinted with permission from ref. 63.

Unlike other lamellar substances, where intercalating species enter the interlayer galleries sporadically, graphite can form intercalation compounds of varying and well-defined structures; this phenomenon is called staging. Intercalation compounds have a large spread of composition as the percentage of intercalated material changes by regular steps as shown in Fig. 3. In the first stage, intercalation reaches a maximum and the material is considered stoichiometric and is known as a first-stage compound.^88^

Intercalation compounds exhibit a wide range of compositions as the proportion of intercalated material varies in regular increments. At the first stage, intercalation reaches its maximum, and the material is deemed stoichiometric, also known as a first-stage compound.^88^ The stage decreases as the concentration of the intercalate increases.^49^

In many cases, reactions occur near ambient temperature, but in general the reaction temperature should be high enough to ensure mobility of the guest species but not so high that the strong bonds are broken and the structure of the host lattice is rearranged. Many intercalation compounds are metastable because of the inherent anisotropy in the chemical bonding.^63^

#### 2.1.2 Thermal behavior of GICs expansion

The initial notable decrease in weight occurs within the temperature range of 150 to 300 °C, which is typically attributed to the evaporation of water (steam) and the release of gases from the more readily detachable functional groups.^9,35^ Botas *et al.* proposed that this phenomenon is caused by a series of events that correspond to the decomposition of oxygen functional groups slightly attached to the carbon structure, beginning with the release of a small amount of water at the initial heating stage and culminating in a dramatic loss at 150–300 °C.^89^ At higher temperatures, specifically in the range of 400 to 950 °C, a gradual and slower decrease in mass occurs. This phenomenon is typically ascribed to the elimination of more stable oxygen functional groups, primarily resulting in the generation of gases such as H_2_ and CO.^9^ Generally, a characteristic where the material has the ability to withstand high temperatures without thermal degradation is thermal stability.^9^

### 2.2 Graphite expansion

Graphite intercalation compounds (GICs), whether they are covalent or ionic type, are generally expandable upon heating. This is because the intercalated species occupy the interlayer spacing, and upon heating, they can expand the distance between the layers of the host graphite lattice. The degree of expansion depends on the type of intercalated species, the amount of intercalation, and the temperature of expansion.^61,86^

Among the different graphite compounds obtained through acid intercalation, graphite bisulfate (graphite-H_2_SO_4_) is widely used to expand and produce EG. This compound is formed by the chemical reaction between graphite, concentrated sulfuric acid, and an oxidizing agent.^34,90^ The reaction between graphite and concentrated sulfuric acid can be represented by the following eqn (1):^36,85,86^1
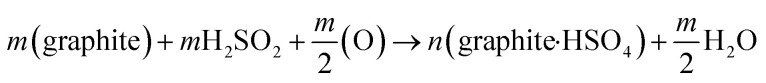
where “O” is the oxidizing agent and the “graphite. HSO_4_” is the GICs which consist of graphite layers intercalated by bisulfate molecules.

The acid molecules penetrate the layers of graphite, and then applying the intercalated graphite to a thermal shock in an electric furnace,^81,91,92^ microwave,^9,50,93^ plasma,^94^ programmable heating,^95^ leads to the rapid vaporization of the intercalated species. The expulsion of these gaseous products generates a force that expands the graphite layers, resulting in a substantial increase in volume. As a result, expanded graphite (EG) with a notably low density is obtained.^72^

Microwave heating is often chosen as a method for expanding graphite intercalation compounds (GICs) because it provides rapid and uniform heating. The unique heating mechanism of microwaves allows for efficient and selective energy transfer to the GICs, leading to their rapid expansion. This uniform heating feature of microwave irradiation contributes to the consistent and controlled expansion of GICs.^9,44^

**Fig. 4:**
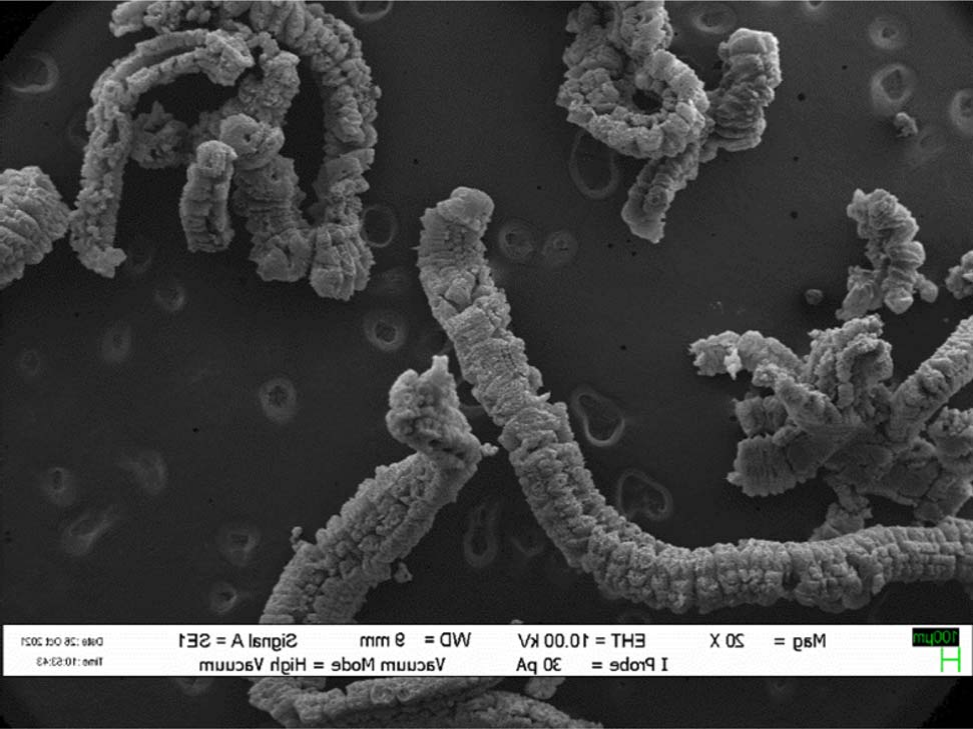
SEM images of expanded graphite. Reprinted with permission from ref. 42.

When exposed to a significant thermal shock up to 1000 °C or excessive heating, such as in a domestic microwave oven, graphite bisulfate undergoes irreversible expansion along the *c*-axis. This expansion can reach hundreds of times the original size. The expansion ratio, which is calculated as the reciprocal of the apparent density, can be remarkably high, reaching values as high as 300. The expanded graphite obtained after the expansion of graphite bisulfate is characterized by its non-compact nature as shown in Fig. 4.

#### 2.2.1 Expansion process

Graphite expansion reactions can be divided into three stages (oxidation, intercalation, and expansion). In the first stage of the graphite expansion process, the oxidant reacts with the edges of the graphite layers, leading to the loss of electrons and the creation of positive charges. These positive charges are then transferred to all the layers of graphite due to the carbon atoms' conjugate structure. The original binding between the layers, which is primarily governed by van der Waals forces, is affected by this transfer of positive charges. As a result, electrostatic repulsion occurs, leading to an increase in the spacing between the layers. In the second stage of the graphite expansion process, the intercalation agent penetrates the interlayer space of the graphite. As it does so, some anions from the intercalation agent are attracted to and adsorbed onto the graphite layers through electrostatic forces. This process contributes to the intercalation of foreign species between the layers of the graphite lattice. At high temperatures, the intercalation agent undergoes decomposition, generating gases as a byproduct. These gases exert a force, known as thrust, that acts to compress the layers along the axial direction of the graphite sheet. This compression leads to the expansion of the graphite, resulting in an increase in its volume.^59,82^

#### 2.2.2 Mechanism of graphite expansion

Intercalation is a complex process where the intercalation process in graphite is chemical as well as physical in nature.^33,49^ Reactions involve adsorption of guest species on host crystals, exchange or insertion at the host surface, the formation of intermediate stages in layered compounds, and transport within the host lattice. Macroscopic effects such as variations in crystal size, dislocations, stacking faults, and pore mouth blockage can strongly influence the kinetics of intercalation.^63^

**Fig. 5:**
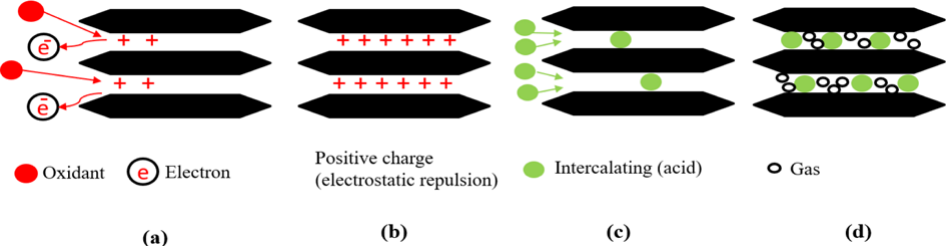
Mechanism diagrams of the complete reaction process. (a) and (b) Oxidation; (c) intercalation; (d) gas release.

Basically, adsorption is a mass transfer process by which a substance is transferred from the liquid phase to the surface of a solid, and becomes bound by physical and/or chemical interactions as schematically exhibited in Fig. 5. It is a partition process in which a few components of the liquid phase are relocated to the surface of the solid adsorbents.^96^ Physical adsorption is accompanied by a decrease in free energy and entropy of the adsorption system and, thereby, this process is exothermic. In graphite adsorption occurs on the planar surface's perpendicular to the *c*-axis as well as on the edge atoms of the carbon planes. Because of the free valence bonds of the edge atoms, the edge atoms tend to be more active. The oxidation reaction is an example of a surface reaction.^49^ The chemicals which are intercalating agent and oxidant agent inserted between graphene layers by adsorption in fluid-to-solid mass transfers which creates a thin film of the acid and oxidant on the graphite.

When the chemicals ions form a chemical bond with atoms of the graphite. As the graphite surface is not electrically neutral, and the atoms are built to bond in three dimensions. In principle, we are considering what happens at a solid–liquid interface. Actually, this ‘surface’ is much more complicated; it allows various types of bonds between ions and molecules on both sides of the solid–fluid interface. At the microscopic level, assuming that equilibrium exists at every point on the fluid–solid interface. Equilibrium here means between the graphite and the concentration of the species in that film. Primarily, this means equality of the chemical potentials on both sides of the liquid–solid interface. Next step is either oxidation and charge transfer, or diffusion of ions and molecules into the lattice or a combination of both processes.^87^ Some ion exchange theories proposed the presence of a thin liquid boundary layer (= film) that covers the solid. The time characterizing ion exchange depends on the relative times of (1) transport of the ions from the bulk solution to the boundary layer and (2) diffusion through the layer. In the case of a porous solid matrix, ions diffuse into the porous solid to adhere on the internal surface of the porous solid. Ion exchange involves also the transport of the released ions back to the bulk solution. The limiting rate is often dictated by the various diffusive steps, rather than by the actual exchange process. Firstly, the oxidant oxidizes the edges of the graphite layers, which causes its electron loss to be positively charged. Due to the conjugate structure of the carbon atoms, these positive charges transfer onto all the layers. The layers are originally bound by van der Waals forces. After the positive charges are transferred, the layer spacing increases because of electrostatic repulsion. Then, the intercalation agent penetrates the interlayer space, and some anions are adsorbed onto the layers through electrostatic attraction.^36,82,97–99^

##### 2.2.2.1 Mechanism of ATEG

**Fig. 6:**
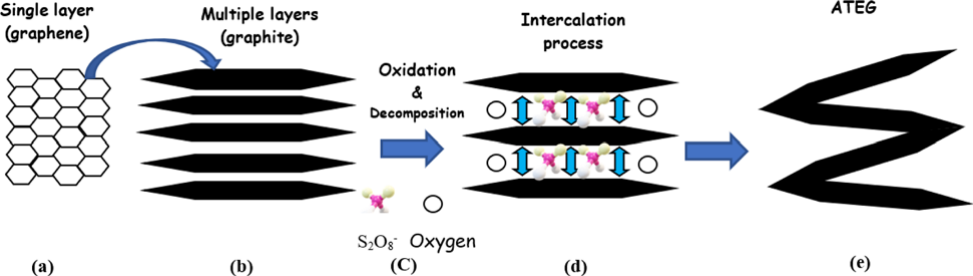
The mechanism scheme of graphite expansion of ATEG (a) single layer (b) multi layers (c) oxidation and decomposition (d) intercalation (e) expansion (worm-like structure). Reprinted with permission from ref. 42.

The original structure of natural graphite flakes was altered to develop a worm-like morphology. Sodium peroxydisulfate decomposition involves the release of gases into graphite interlayer spaces. It is feasible that increasing pressure from gases will be sufficient to overcome the van der Waals forces within the interlayers, resulting in a certain thrust and the effective expansion of graphite in the direction of the *C*-axis.^38^ Therefore, it is concluded that when a binary-component solution containing sodium peroxydisulfate and concentrated sulfuric acid is employed for EG synthesis, portions of sodium peroxydisulfate operate as an oxidant, allowing H_2_SO_4_ to intercalate into the gallery of graphite interlayers. During the intercalation process of H_2_SO_4_, some of the excessive sodium peroxydisulfate may be drawn into the graphite's layers gallery. Chemically, the sodium peroxydisulfate forming an unstable compound when it reacts with the high concentration H_2_SO_4_.^100^ Due to the chemical instability in an acidic medium, sodium peroxydisulfate may decompose and generate O_2_ gas, causing an instantaneous pressure rise within the graphite interlayer gallery, causing graphite to expand. As the released gas is responsible for the graphite expansion, which raises the instantaneous pressure, it should generate more gas to get a larger expansion rate as shown in Fig. 6.

Nevertheless, the mechanism behind this efficient process is yet understood entirely, and more research is needed. According to some studies, S_2_O_8_^2−^ is strongly oxidizable and unstable in acidic medium, causing it to decompose and release oxygen.^39,100,101^ The proposed and modified reaction mechanisms of the decomposition for Na_2_S_2_O_8_ mixed with the concentrated H_2_SO_4_ could be expressed as the following eqn (2)–(5):

2 S_2_O_8_^2−^ + H_2_O → H_2_SO_4_^−^ + ½O_2_

3 H_2_S_2_O_8_ + H_2_O → H_2_SO_5_ + H_2_SO_4_

4 H_2_SO_5_ + H_2_O → H_2_O_2_ + H_2_SO_4_

5
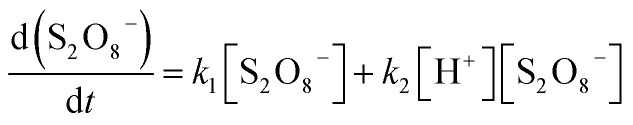
The values of *k*_1_ and *k*_2_, respectively, are 6.0 × 10^−5^ m K^−1^ and 3.5 × 10^−3^ (min^−1^) (m L^−1^)^−1^ at an ionic strength of 0.4 at 50 °C.^39^

The H_2_SO_4_^−^ shifted to H_2_S_2_O_8_ and H_2_O. The H_2_S_2_O_8_ shifted to S_2_O_8_^2−^ and H_2_O in a moment.^38,100^

The (S_2_O_8_^2−^) can be thermally activated to produce a powerful oxidant known as the sulfate free radical (SO_4_^2−^).^102^

6 S_2_O_8_^2−^ → 2SO_4_^2−^

7 2SO^−^_4_ + 2H_2_O → 2HSO^−^_4_ + 2HO

8 2HO → H_2_O + ½O_2_

The standard oxidation–reduction potential for the reaction is 2.1 V, compared 1.8 V for hydrogen peroxide (H_2_O_2_) and 1.4 V for the peroxy-monosulfate anion (HSO_5_^−^). This potential is greater than that of the permanganate anion (MnO_4_^−^), which is 1.7 V, but slightly less than that of ozone, which is 2.2 V.^103^

Mixing Na_2_S_2_O_8_ with H_2_SO_4_ could result in a violent heat decomposition and pressure rise. Attention was focused on the Na_2_S_2_O_8_ forming an unstable compound when it reacted with the high concentration of H_2_SO_4_, which had a great quantity of H_2_SO_4_ and with increasing the mass of Na_2_S_2_O_8_ shifted quickly to Na_2_S_2_O_8_ decomposition reaction.

##### 2.2.2.2 Mechanism of HTEG

**Fig. 7:**
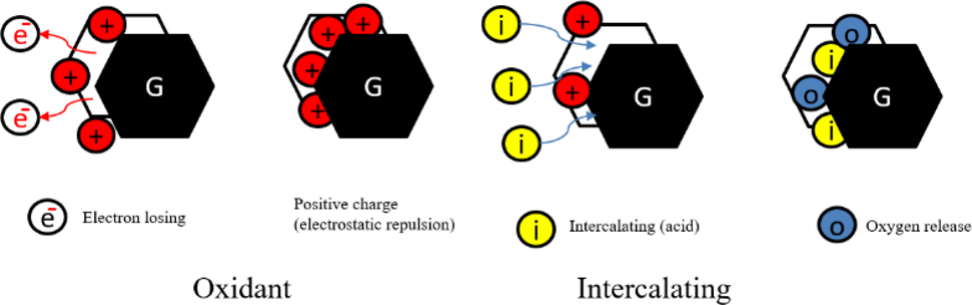
The mechanism scheme of graphite expansion of HETG.

The expansion of expandable graphite results from disjoining pressure which is formed during its heating process. The process as shown in Fig. 7 may be simply modeled as: intercalating species in expandable graphite can be considered as a liquid or solid phase which is fixed between the graphite sheets. Heating expandable graphite leads to conversion of the intercalating species from a liquid or solid phase to a gas phase. The formation of gas results in an increase in volume of the intercalating species of about 1000-fold. The pressure formed by this volume increase forces the adjacent graphite layers to separate, thereby resulting in the expansion of expandable graphite.^38,104^ Therefore, the expansion ratio is affected strongly by the disjoining pressure which is derived from the gas. In order to increase expansion ratio, therefore, we should prevent gas from escaping through the edges of graphite flakes.^105^ The mechanism of graphite expansion at the micro-level can be described as follows:

In the initial stage, the oxidant agent causes oxidation of the edges of the graphite layers, resulting in the loss of electrons and the generation of positive charges. These positive charges are transferred to all layers due to the conjugate structure of carbon atoms. Graphite intercalation compounds (GICs) then enter the interlayer space, and certain anions are attracted to and adsorbed onto the layers through electrostatic attraction. During the decomposition of the GICs, gaseous products are formed. These gases create high pressure between the interlayer graphene sheets, exerting a force that pushes the graphite layers apart. The release of gases generates a driving force between the graphite sheets. This driving force overcomes the van der Waals forces that hold the graphite sheets together, promoting the expansion of the particles from the edges towards the center of the sheets.^82^

### 2.3 Characterization

The expanded graphite has a unique structure and properties. Therefore, identifying crystal structures and functional groups is essential for future applications. There are different characterization techniques for the identification of expanded graphite. In this regard, the structures of the natural graphite flakes and expanded graphite samples were analyzed by X-ray diffraction (XRD, Shimadzu, XED 6000).

X-ray diffraction (XRD) analysis is a widely used technique to determine the crystal structure and properties of materials. It provides information about the arrangement of atoms or molecules in a material by analyzing the diffraction pattern produced when a crystalline sample is exposed to X-rays. The basic principle behind XRD analysis is that when X-rays interact with a crystal, they undergo constructive interference, resulting in the generation of a diffraction pattern. This pattern consists of a series of distinct spots or peaks corresponding to the scattering of X-rays by the crystal lattice planes.^34,63^

In graphite expansion, X-ray diffraction (XRD) analysis can be used to investigate the expansion of graphite through measurements of its crystal structure. When graphite undergoes expansion, the interlayer spacing between the graphene layers increases. This change in interlayer spacing can be detected and quantified using XRD. By analyzing the XRD data, it is possible to determine the interlayer spacing in the expanded graphite and quantify the extent of expansion. This information can be used to study the effects of different processing conditions or intercalating agents on the expansion behavior of graphite. XRD analysis provides valuable insights into the structural changes occurring in graphite during expansion, allowing for a better understanding of its properties and potential applications.^34,106^

The surface functional groups of EG were analyzed by Fourier transform infrared spectroscopy (Nicolet FT-IR 6700, Thermo Fisher Scientific). Fourier transform infrared (FTIR) analysis is a widely used spectroscopic technique that provides information about the chemical composition and molecular structure of a sample. It involves the measurement of the absorption, transmission, or reflection of infrared radiation by the sample. The basic principle behind FTIR analysis is that different chemical bonds within a molecule vibrate at specific frequencies, known as vibrational frequencies, in the infrared region of the electromagnetic spectrum. By measuring the absorption or transmission of infrared light across a range of frequencies, FTIR spectroscopy can identify the functional groups present in a sample and provide information about molecular structure, chemical bonding, and the presence of specific compounds.^107^

FTIR analysis can be used to investigate the expansion or intercalation of graphite by studying the changes in its infrared absorption spectrum. Specifically, in the context of graphite expansion, FTIR analysis can provide information on the intercalation of molecules or ions between the graphene layers. It can detect the presence of intercalated species by changes in absorption bands related to the vibrational modes of the intercalated molecules or ions. By comparing the FTIR spectrum of expanded graphite with that of the pristine graphite, one can identify new peaks or changes in peak intensity, which indicate the presence of intercalated species. The analysis can also provide insights into the type of intercalated species and the bonding interactions between them and the graphite layers. FTIR analysis is a valuable tool to study the structural changes in graphite due to intercalation or expansion and can help in understanding the intercalation mechanisms, optimizing intercalation processes, and developing new functional materials based on expanded graphite.^34,106^

The micro-morphologies were viewed by field emission scanning electron microscopy (LEO 1445 VP scanning electron microscope). Generally, SEM analysis is a powerful imaging technique used to examine the surface morphology and topography of materials at high magnification. It provides detailed information about the sample's surface features, such as shape, size, texture, and composition. It provides valuable insights into the microstructure and surface properties of materials, allowing researchers and scientists to study, understand, and optimize material behavior and performance.^34^

SEM analysis can be used to examine the surface morphology and structural changes in graphite due to expansion or intercalation. SEM analysis provides valuable visual information about the surface structure and morphology of expanded graphite. It can help in understanding the effects of expansion processes, intercalation agents, or other factors on the graphite structure.^9,34^

## 3. Thermally expanded graphite preparation

TEG, or thermally expanded graphite, is a type of graphite that has been thermally processed to increase its volume and create a highly porous structure. Some of the main features of TEG include a highly porous structure: TEG has a very open, sponge-like structure, which makes it useful in applications where high surface area is desired. Lightweight: TEG has a very low apparent density, typically between 0.002 and 0.02 g cm^−3^, which makes it an attractive material for lightweight applications. High mechanical properties: despite its low density, TEG has excellent mechanical properties, with a tensile strength of around 10 MPa. Thermal conductivity: TEG has high thermal conductivity, typically between 25 and 470 W m^−1^ K^−1^, making it useful in applications where heat needs to be transferred efficiently. High electrical conductivity: TEG also has high electrical conductivity, typically between 106 and 108 S m^−1^, making it useful in electrical applications. Low-cost: TEG is relatively inexpensive to produce compared to other high-performance materials, making it attractive for cost-sensitive applications.^59,86^

**Fig. 8:**
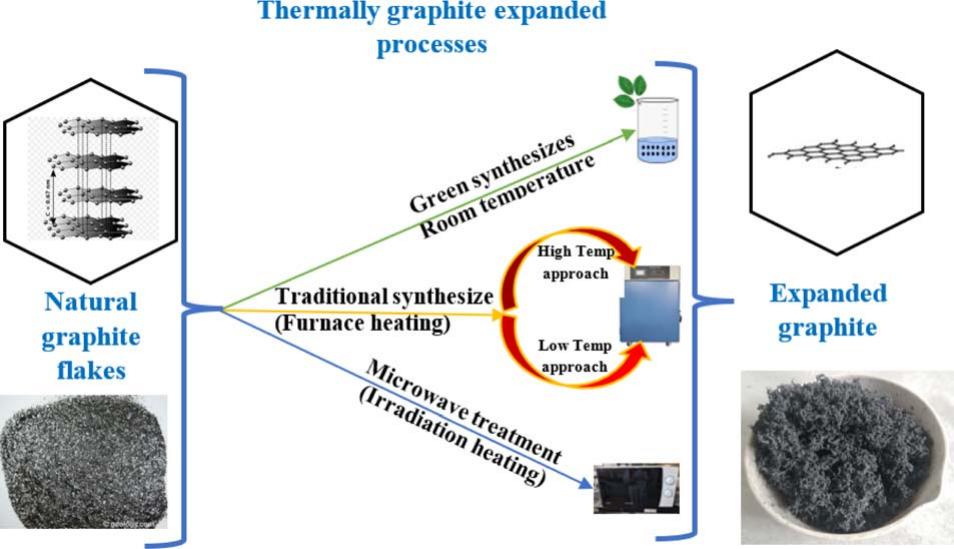
Graphite expansion techniques.

Numerous researchers have dedicated their efforts to investigating different preparation methods for expanded graphite. They have explored various factors such as the choice of oxidant agent, intercalation agent, and heating duration in order to achieve specific goals such as maximizing surface area or minimizing bulk density. The objective is to find the optimal conditions that result in expanded graphite with desirable properties. In general, researchers have found that optimum conditions for preparing expanded graphite involve using either muffle or tubular furnaces with a temperature of around 900 °C or alternatively, irradiation techniques such as microwave heating have also been explored, with a heating time ranging from 5 to 30 seconds for both techniques. These conditions have been identified as effective in achieving the desired expansion and properties of the graphite material.^59,72^ These methods provide alternative options depending on the desired degree of expansion, scalability, and specific properties required for particular applications as shown in Fig. 8 below.^108^

### 3.1 Expansion of graphite at room temperature

The impact of energy requirements on the overall cost of the expansion process is widely recognized. Methods that require high temperatures or extensive heating may consume more energy and increase operational costs. Energy-efficient preparation methods can help minimize costs and improve the economic viability of the process.

**Fig. 9:**
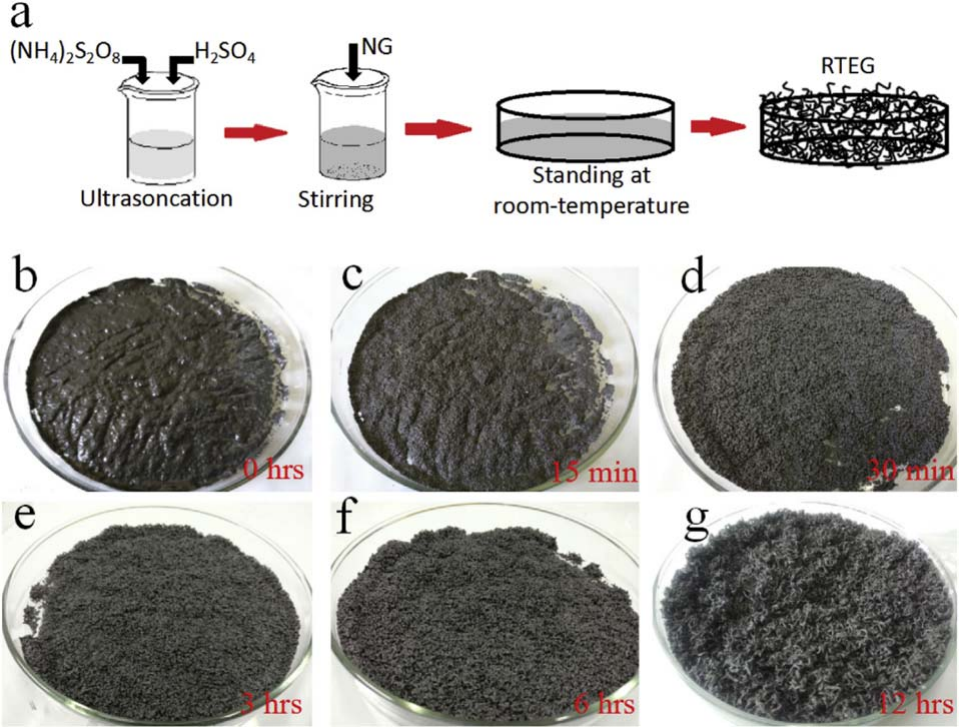
(a) The schematic diagram of thermally synthesis by the one-step room-temperature method. (b–g) Optical images of the graphite intercalation and morphological evolutions with time from 0 to 12 h. Reprinted with permission from ref ^58^.

Liu *et al.*^58^ presented a one-step method for the production of expanded graphite at room temperature as illustrated in Fig. 9. In this method, the graphite material was intercalated by using concentrated sulfuric acid and ammonium persulfate as the intercalating agents. The process involved the addition of the intercalating agents to the graphite, followed by sonication stirring to facilitate the mixing and intercalation process. Moreover, the amount of oxidant considered high compared to the intercalating agent and the graphite. The resulting expanded graphite exhibited a specific volume expansion of 225 mL g^−1^, indicating a significant increase in volume compared to the original graphite material. However, it is important to note that this process did not involve any washing steps, which means that the expanded graphite produced may require additional treatment or purification steps before it can be used in applications. The one-step method described by Liu *et al.*^58^ offers a convenient approach for the production of expanded graphite, but the use of special techniques such as sonication stirring and the relatively high amount of oxidant used may have certain implications for the overall process and the quality of the final product.

In a recent study by Ardestani *et al.*,^109^ expanded graphite was successfully developed with an expansion volume of 250 mL g^−1^ at room temperature. The process involved the use of a tricomponent system comprising potassium permanganate, sulfuric acid, and hydrogen peroxide for the reaction and intercalation of flake graphite. While the study provided details about the reaction and intercalation steps, it did not specifically mention the drying process for the resulting graphite intercalation compounds (GICs). The entire process, from GIC preparation to graphite expansion, took more than two days, indicating that it required a considerable amount of time. This extended duration is likely due to the need for sufficient intercalation and reaction time to achieve the desired expansion volume. Finally, the study by Ardestani *et al.* demonstrated the successful production of expanded graphite at room temperature using a tricomponent system. However, further information regarding the drying process and the specific conditions used for intercalation and expansion would be required for a more comprehensive understanding of the methodology.

### 3.2 Expansion of graphite at low temperature

**Fig. 10:**
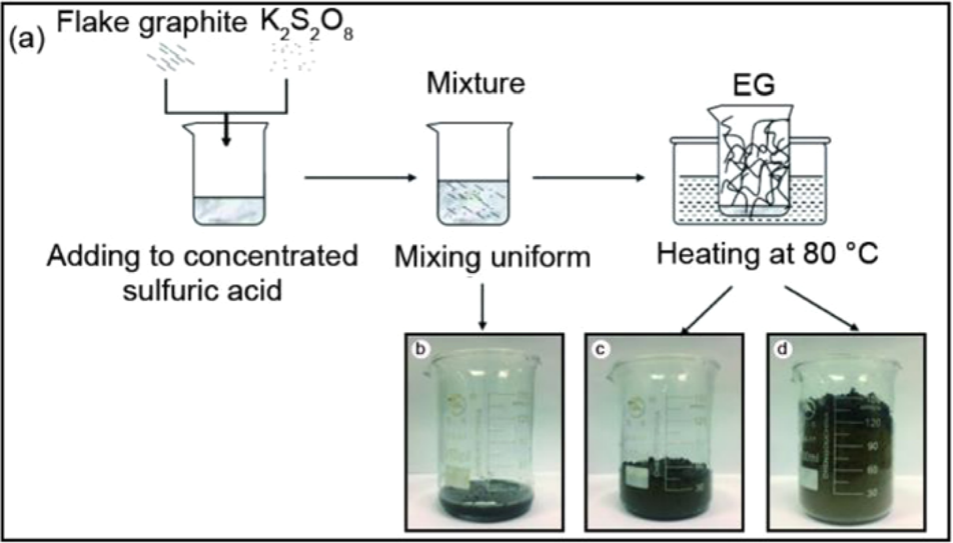
(a) An illustration of thermally synthesis of EG, (b) heating for 0–30 s, (c) heating for 1 min, and (d) heating for 2 min at 80 °C. Reprinted with permission from ref ^38^.

Graphite expansion at low temperatures refers to the process of inducing the expansion of graphite at temperatures below the typical high-temperature range used in thermal expansion methods. As presented in Fig. 10 the low-temperature expansion allows for the modification of graphite without subjecting it to extreme heat, providing certain advantages in terms of energy consumption and process control. These low-temperature expansion techniques offer advantages such as reduced energy consumption, enhanced process control, and the ability to modify graphite without compromising its structure or properties. However, it's important to note that the specific parameters, intercalating agents, and conditions required for low-temperature graphite expansion can vary depending on the desired degree of expansion and the targeted application.^25,38^

S. Hou and his team^110^ successfully prepared expanded graphite (EG) blocks using ultra-large flakes of graphite. They achieved this by intercalating the graphite with H_2_SO_4_ and utilizing a significant amount of H_2_O_2_ as an intercalating agent. The intercalation process involved reaching a maximum temperature of up to 80 °C. Subsequently, the intercalated graphite underwent expansion within a closed container, resulting in the successful preparation of EG blocks with bulk densities ranging from 0.008 to 0.024 g cm^−3^. S. Hou, S. He, T. Zhu, and other researchers^25^ proposed an environmentally friendly method for preparing expanded graphite using a binary system of sulfuric acid and hydrogen peroxide. Through this method, they were able to achieve an expansion volume of up to 320 mL per gram at a temperature of 60 °C for a duration of 4.5 to 9.5 hours. Bo Hou *et al.*^38^ prepared EG using concentrated sulfuric acid and potassium persulfate. The process involved heating the mixture at 80 °C for 5 minutes, followed by vacuum filtration. The resulting material was then dried in an oven at 60 °C for 5 hours, resulting in the production of expanded graphite. The volume of EG obtained through this method reached 150 mL per gram. G. Zhao and his team^111^ employed a multi-step process to obtain expanded graphite with a 10-fold expansion volume. Before usage, they effectively eliminated moisture from the graphite through vacuuming and drying. Furthermore, they employed a sophisticated technique involving four chemicals and numerous intricate steps. J. Huang *et al.*^74^ produced expandable graphite with an expansion volume of 282 mL per gram. This was achieved by utilizing two intercalating agents, sulfuric acid and phosphoric acid, along with hydrogen peroxide. The process took approximately 15 hours at a temperature of 60 °C.

### 3.3 Expansion of graphite at high temperature

**Fig. 11:**
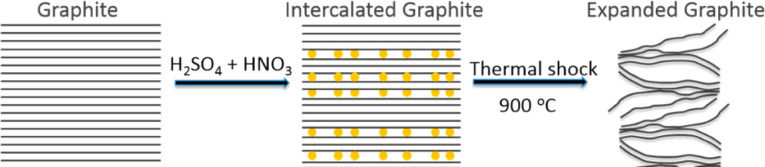
Schematic showing the expansion of graphite at high temperature. Reprinted with permission from ref ^114^.

Graphite expansion at high temperatures refers to the process of inducing the expansion of graphite by subjecting it to elevated temperatures as shown in Fig. 11. This high-temperature treatment allows for the modification of graphite by promoting the separation and expansion of the graphene layers within the structure. Graphite is heated to temperatures typically exceeding 1000 °C in a controlled environment. As the temperature increases, the thermal energy disrupts the interlayer bonding forces, causing the graphene layers to separate and expand. The expansion can be controlled by adjusting the temperature and duration of heating.^112,113^ The high-temperature expansion methods provide greater control over the degree of expansion and allow for modifications to the graphite structure. However, it's important to note that the specific parameters, heating rates, and temperature ranges required for high-temperature graphite expansion can vary depending on the desired outcome and application.^81^

G. Yin *et al.* (2021) produced expanded graphite at an expansion temperature of 900 °C. The specific surface area of EG was found to be approximately 120 times larger than that of NFG (non-expanded graphite). Additionally, they observed that the pores in EGs were predominantly distributed within the pore size range of 3.0–10 nm. This indicates that the EGs prepared in their study can be classified as mesoporous carbon materials.^91^ Bannov *et al.* (2021) synthesized expanded graphite using the programmable heating technique, employing heating with a constant rate of 20 °C min^−1^ from room temperature to 400–700 °C. They compared this method with thermal shock as methods of producing expanded graphite and found that the programmable heating technique demonstrated greater efficiency, particularly at a temperature of 400 °C. The resulting expanded graphite exhibited surface areas of 699 and 184 m^2^ g^−1^, respectively. The researchers also discovered that their novel method of production, involving intercalated graphite, enabled them to achieve a relatively higher yield ranging from 78% to 90%.^95^ Nirwan *et al.* (2021) conducted a study where they prepared expanded graphite utilizing graphite flakes with a mesh size of +100. They employed KMnO_4_ as an oxidizing agent and H_2_SO_4_ as intercalates. Under optimized conditions, they achieved a high expansion volume of 375 mL per gram and a good surface area of 100 m^2^ per gram. The optimized ratios of graphite flakes, KMnO_4_, and H_2_SO_4_ were 1 : 0.15 : 2.5, respectively, with a 65% mass concentration. The reaction time was optimized to 3 hours.^92^ K. H. Wu *et al.* (2021) conducted a study to produce expanded graphite with a high expansion volume. They used a mixture of sulfuric acid and nitric acid as the intercalating agents and natural flake graphite as the starting material. The intercalation process involved mixing the acids with the graphite for a duration of 4 hours. This allowed the acids to penetrate between the layers of the graphite and form graphite intercalation compounds (GICs). Subsequently, the GICs were dried for 24 hours at a temperature of 60 °C to remove any residual moisture. Finally, the GICs heated up to 1150 °C to obtain the expanded graphite.^19^ Çalın *et al.* (2020) conducted an investigation to examine the relationship between the expansion ratio and the pore structure of expanded graphite. They achieved this by modifying the expansion conditions of expandable graphite with different flake sizes. The highest expansion ratio of 80 mL g^−1^ was obtained for the 10-mesh sample when exposed to a temperature of 1150 °C. In contrast, the +50 mesh (>300 μm) commercial expandable graphite demonstrated an expansion ratio of 400 mL g^−1^ at the same temperature (1150 °C) within a closed-lid crucible.^81^ Wang *et al.* (2020) carried out the preparation of expanded graphite through a series of steps. Initially, they converted graphite into intercalated graphite by subjecting it to chemical oxidation using a mixture of sulfuric and nitric acid. The resulting intercalated graphite was then dried in a vacuum oven at 65 °C for 24 hours. Subsequently, it underwent heat treatment at 900 °C for 60 seconds. The porous graphite sheets obtained from this process were further compressed to form bulk expanded graphite. The density of the bulk expanded graphite was increased by applying higher pressure.^115^ Z. Liu *et al.* (2018) produced expanded graphite with an expansion ratio of up to 100 times that of ordinary graphite. They achieved this by utilizing a rarely large flake graphite, specifically a 9-mesh graphite.^116^ Tingkai Zhao *et al.* (2018) produced expanded graphite through a series of steps, including stirring, filtering, washing, and drying. Subsequently, they achieved the expansion of graphite by subjecting it to rapid expansion in a muffle furnace at a temperature of 800 °C for a duration of 10 seconds.^79^ Peng *et al.* (2018) conducted a study on the preparation of expandable graphite at a low temperature of 400 °C. They utilized natural flake graphite as the raw material and employed potassium permanganate, perchloric acid, and ammonium nitrate as oxidative intercalants. Through the oxidation and intercalation process, the intercalation reagents introduced oxygen-containing groups onto the edges and within the layers of the graphite product. This led to an expansion of the interlayer spacing while preserving the inner hexagonal structure of the graphite.^97^ Trivedi *et al.* (2018) made an observation regarding the oxidation of graphite by potassium permanganate in concentrated sulfuric acid compared to a potassium dichromate-concentrated sulfuric acid mixture. They found that potassium permanganate in concentrated sulfuric acid more efficiently oxidizes the sp^2^ carbon atoms in graphite, resulting in the formation of graphite bisulfate. Furthermore, they investigated the impact of varying heat treatment temperatures ranging from 400 °C to 1030 °C on expandable graphite treated with KMnO_4_ in concentrated sulfuric acid and graphite treated with K_2_Cr_2_O_7_ in concentrated sulfuric acid. Their findings indicated that the expandable graphite treated with KMnO_4_ in concentrated sulfuric acid exhibited superior expansion compared to the graphite treated with K_2_Cr_2_O_7_ in concentrated sulfuric acid.^77^ J. He *et al.* (2017) successfully prepared sulfur-free expanded graphite using a two-step chemical oxidation process. They utilized natural flake graphite as the precursor material. The resulting expanded graphite exhibited an expansion volume of 420 mL g^−1^.^17^ Ting Yao *et al.* (2016) synthesized macroscopic expanded graphite, which has the potential for large-scale development. The expanded graphite exhibited unique physical characteristics, including low apparent density, high porosity, excellent mechanical stability, and high hydrophobicity.^53^

### 3.4 Expansion of graphite *via* microwave irradiation

Preparation of expanded graphite using the microwave irradiation technique involves subjecting graphite material to microwave radiation, which induces thermal energy and leads to the expansion of the graphite structure.^93^ The microwave irradiation technique offers advantages such as rapid heating, uniform heating distribution, and potential energy efficiency compared to traditional thermal methods. It allows for the preparation of expanded graphite with unique structural and morphological properties.^59,108^ The preparation of expanded graphite at high temperatures and the use of microwave irradiation as a technique have some key differences:

(1) Heating mechanism: in high-temperature preparation, heat is typically provided by conventional thermal sources such as ovens or furnaces, while microwave irradiation utilizes electromagnetic waves to directly generate heat within the material.^117^

(2) Heating rate: high-temperature preparation usually involves slower heating rates to ensure controlled expansion, while microwave irradiation allows for rapid and selective heating, leading to faster expansion.^9,52^

(3) Energy efficiency: microwave irradiation is generally more energy-efficient than high-temperature methods. Microwaves directly heat the intercalated graphite, reducing energy loss to the surrounding environment.^59^

(4) Expansion control: high-temperature methods provide better control over the expansion process due to the ability to adjust and monitor temperature parameters. Microwave irradiation, on the other hand, may result in faster and sometimes less controllable expansion due to rapid and intense heating.^9^

(5) Scale and throughput: high-temperature methods are often more suitable for large-scale production due to the availability of larger furnaces and ovens. Microwave irradiation, although capable of producing expanded graphite, may have limitations in terms of batch size and throughput.^38^

(6) Morphology and properties: the differences in heating mechanisms and rates can influence the morphology and properties of the expanded graphite. High-temperature methods tend to produce more uniform and controlled expansion, while microwave irradiation may result in a more heterogeneous structure with potentially different properties.^81^

**Fig. 12:**
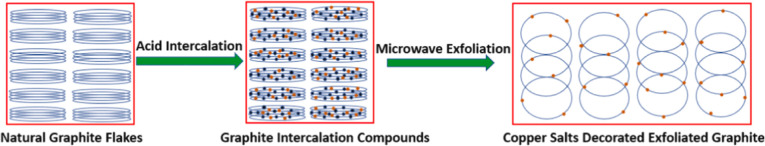
Schematic representation of the expansion of graphite *via* microwave technology. Reprinted with permission from ref ^93^.

The choice between high-temperature preparation and microwave irradiation depends on factors such as desired expansion control, energy efficiency, scale of production, and specific application requirements. Both methods have their advantages and limitations, and the selection depends on the specific goals of the preparation process.^51^ Microwave irradiation is a more promising method compared to conventional heating for several reasons. Firstly, it can be conducted at room temperature, eliminating the need for high-temperature environments. Secondly, microwave irradiation significantly reduces the processing time, allowing for faster production. Lastly, this method consumes less energy, making it a more energy-efficient option. Overall, the use of microwave irradiation offers advantages such as reduced processing time, lower energy consumption, and the ability to operate at room temperature, making it a favourable choice for the preparation of expanded graphite.^51,54^ Saikam *et al.*^93^ used microwave irradiation to expand graphite as shown in Fig. 12.

Gabriela Tarango-Rivero *et al.* (2022) focused on developing alternative chemical routes for producing expanded graphite with reduced environmental impact, based on the principles of green chemistry. They proposed a process for expanded graphite production that employs only two intercalation chemicals, significantly reducing the consumption of sulfuric acid to just 10%. Additionally, they eliminated the use of strong oxidant salts, which are known to have negative environmental effects. During their study, the team evaluated three key process parameters: the effect of milling, peroxide concentration, and microwave expansion. These parameters were carefully examined to optimize the production process and achieve the desired expansion of the graphite material. The research carried out by Gabriela Tarango-Rivero and her team demonstrates a commitment to developing more sustainable methods for expanded graphite production, considering the principles of green chemistry and minimizing the environmental impact associated with traditional production approaches.^9^ Siakam *et al.* (2022) developed EG by oxidizing natural graphite with perchloric acid and cupric nitrate. The mixture was stirred at ambient temperature for 20 s. During the mixing step, perchloric acid was intercalated between the layers of graphite, resulting in the formation of graphite intercalation compounds (GICs). After that, the mixture was transferred to a microwave oven and irradiated for 40 s at 800 W. The increase of the graphite layers was noticed at this time, along with fuming and lightning effects. SEM investigation revealed the worm-like structure of EG, and the surface morphology of EG revealed a well-expanded structure with expansion occurring along the direction of the *c*-axis.^93^ Zhen-Xue Liu *et al.* (2022) synthesized expanded graphite using two different chemical methods but the same technique. They referred to this expanded graphite as “SEG” or spitball expanded graphite. It is noteworthy that the two types of SEG exhibited significant differences compared to traditional expanded graphite. They lacked the characteristic worm-like structures and web-like pores typically found in traditional expanded graphite. However, these unique properties make SEG suitable as user-friendly, non-polar adsorbents in various engineering applications that do not rely on a homogeneous structure. SEG can be utilized for purposes such as adsorption or separation of aromatic compounds and graphene production.^113^ Zhen-Xue Liu *et al.* (2020) conducted a study where they produced a slightly expanded graphite with an expansion ratio of only four times. The researchers used a microwave treatment with a power of 1000 W for 2 minutes, which is considered the highest microwave power used thus far in similar studies. The preparation method they employed involved an improved version of the Hummers' method, which aimed to convert natural flake graphite into expanded graphite. The process involved a sophisticated procedure and the use of excessive chemicals, resulting in only a slight expansion volume being achieved.^54^ Van Pham *et al.* (2019) synthesized EG in two steps. First, graphite fakes, potassium permanganates, acetic anhydride, and perchloric acid were mixed for a few seconds in a ratio of 1 : 0.5 : 1 : 0.4 (g g^−1^), and the mixture was placed in a microwave oven at 360 W for 50 seconds to generate graphite sheet expansion.^118^ Ngoc Bich Hoang *et al.* (2019) focused on the production of sulfur-free expanded graphite. They employed a specific method utilizing perchloric acid as a key component. The expansion of graphite was achieved through the utilization of potassium permanganate as an oxidizing agent and perchloric acid as an intercalating agent. The process was facilitated by the application of a microwave-assisted technique. This approach allowed for the production of sulfur-free expanded graphite, which can have potential applications in various fields.^45^ Tianyu Zhou *et al.* (2018) developed a one-step synthesis process for the production of boron acid-modified expanded graphite (B-EG) with the assistance of microwave irradiation. The process involved the use of H_2_SO_4_ and H_3_BO_3_ as intercalating agents, while KMnO_4_ served as the oxidizing agent. The resulting B-EG exhibited a unique structure characterized by a combination of worm-like and lamellar morphology, along with a significant presence of small dispersed particles throughout the material.^51^ Sykam, Jayram, and Rao (2018) conducted a study on the rapid and cost-effective production of expanded graphite using microwave irradiation and a binary system of NFG (natural flake graphite) and perchloric acid. The process involved intercalating the graphite flakes with perchloric acid, which quickly entered the layers and formed graphite intercalation compounds (GICs). The GICs were then rapidly expanded using microwave heating, resulting in the formation of highly porous EG materials with a unique worm-like structure. The expansion volume achieved was 524 mg L^−1^, and the entire process took approximately one minute (60–70 seconds) to complete.^60^ Fengshuang Zhang *et al.* (2015) introduced a rapid and efficient method for the preparation of expanded graphite. They utilized microwave irradiation to accelerate the expansion process, resulting in a significantly shortened preparation time. In fact, the entire preparation process could be completed in less than 4 minutes, allowing for faster and more efficient production of expanded graphite.^52^ N. Sykam and K. Kar (2014) successfully produced expanded graphite in a rapid manner using microwave irradiation techniques. In their study, they utilized perchloric acid as the intercalation agent. Perchloric acid is known to be a stronger oxidant compared to sulfuric acid, which is commonly used in traditional methods. However, it is important to note that perchloric acid is more challenging to handle and requires careful precautions due to its hazardous nature. Therefore, while the microwave irradiation method offers the advantage of rapid EG production, it also poses some risks due to the use of a stronger and potentially more hazardous intercalation agent. Proper safety measures and handling protocols should be followed when employing this method.^44^ J. Kim *et al.* (2014) conducted a study to examine the expanding characteristics of graphite in microwave-assisted expansion. They investigated several factors that influence the expansion process, including the mixing ratio of graphite, KMnO_4_, and HNO_3_; the mixing time; and the characteristics of the graphite, such as its form (natural *vs.* synthetic), type (lump *vs.* flake), and size (10 to 40 μm in diameter). Thus, this study contributes to the understanding of the microwave-assisted expansion of graphite and provides valuable insights into the factors affecting the expansion process and the resulting chemical composition of the expanded graphite samples.^117^

### 3.5 Expansion methods' summary

Table 2 summarizes the previous and recent representative studies on the thermally synthesis of expanded graphite and their application.

**Table 2:** Summary of graphite expansion methods^a^

Strarting materials	*T* (°C) or IR (W)	Time	EV (mL g^−1^) or density (g cm^−3^)	Application	Ref.
NGF (50 m): H_2_SO_4_ (i): (NH_4_)_2_S_2_O_8_ (ox)	RT	*t*_i_ = 5 min, *t*_s_ = 12 h	225 mL g^−1^	—	58
NGF (100 m): H_2_SO_4_ (i): KMnO_4_: H_2_O_2_ (ox)	RT	*t*_i_ = 25.5 h, *t*_d_ = —	250 mL g^−1^	Cationic dye removal	109
NGF: H_2_SO_4_(i): H_2_O_2_ (ox)	*T*_i_: 5 °C, *T*_d_: 80 °C	*t*_i_ = 30 min, *t*_s_ = 10 h, *t*_d_ = 2 h	0.008–0.024 g cm^−3^	Oil sorption	110
NGF (300 μm): H_2_SO_4_ (i): H_2_O_2_ (ox)	*T*_i_: 5 °C, *T*_s_: 40 °C, *T*_d_: 60 °C	*t*_i_ = 30 min, *t*_s_ = 4 h, *t*_d_ = 5 h	320 mL g^−1^	Oil sorbents	25
NGF (300 μm): H_2_SO_4_ (i): K_2_S_2_O_8_ (ox)	*T*_i_: 80 °C, *T*_d_: 60 °C	*t*_i_ = 5 min, *t*_d_ = 5 h	150 mL g^−1^	Recommended use is graphene preparation	38
NGF (300 μm): HClO_4_ (i): NH_2_SO_3_H: NaNO_3_: KMnO_4_: H_3_PO_4_: NaOH (ox)	*T*_i_: 40 °C, *T*_d_: 60 °C	*t*_i_ = 90 min, *t*_d_ = 6 h	10 times	Steam channelling control in heavy oil reservoirs	111
NGF (50 m): HClO_4_ (i): H_2_O_2_: KMnO_4_: HAc (ox)	*T*_i_: *T*_r_, *T*_s_: RT & 40 °C, *T*_d_: 60 °C	*t*_i_ = 12 min, *t*_s_ = 6, 1 h, *t*_d_ = 8 h	273 mL g^−1^	Steam plugging agent in heavy oil reservoirs	82
NGF (300 μm): H_2_SO_4_ (i): H_3_PO_4_: H_2_O_2_ (ox)	*T*_i_: 40 °C, *T*_d_: 65 °C	*t*_i_ = 3 h, *t*_d_ = 12 h	282 mL g^−1^	Flame-resistance	74
NGF: H_2_SO_4_ (i): HNO_3_ (ox)	*T*_i_: RT, *T*_d_: 60 °C, 1150 °C	*t*_i_ = 4 h, *t*_d_ = 24 h	300 mL g^−1^, 34.8 m^2^ g^−1^	Oil sorption	19
NFG (50–60) m: HClO_4_ (i): K_2_Cr_2_O_7_: CH_3_COOH: HNO_3_ (ox)	*T*_i_: RT, *T*_d_: 80 °C, 900 °C 5 s	*t*_i_ = 40 min, *t*_d_ = 40 min	120 mL g^−1^, 124 m^2^ g^−1^	Adsorption	91
NGF (+100) m: H_2_SO_4_ (i): KMnO_4_ (ox)	*T*_i_: RT, *T*_s_: 120 °C, 900 °C	*t*_i_ = 3 h, *t*_s_ = overnight	375 mL g^−1^	Oil spills application	92
NGF (+50 and 10) m: H_2_SO_4_ (i): HNO_3_ (ox)	*T*_i_: RT, *T*_d_: 100 °C, 1150 °C 2 min	*t*_i_ = 130 min, *t*_d_ = 24 h	400 mL g^−1^, 4.63 m^2^ g^−1^, 80 mL g^−1^, 41 m^2^ g^−1^	Study different flake sizes on EG production	81
NGF: H_2_SO_4_ (i): HNO_3_ (ox)	*T*_i_: —, *T*_d_: 65 °C, 900 °C 60 s	*t*_i_ = —, *t*_d_ = 24 h	0.21 g cm^−3^	Phase change materials	115
NGF >1.25 mm: HClO_4_ (i): H_2_O_2_ (ox)	*T*_i_: RT, *T*_d_: 80 °C, 1100 °C 60 s	*t*_i_ = 50 min, *t*_d_ = —	178.3 mL g^−1^, 100.97 m^2^ g^−1^	Oil sorption	118
NGF (+100) m: H_2_SO_4_ (i): KMnO_4_: K_2_Cr_2_O_7_ (ox)	*T*_s_: RT, *T*_d_: 120 °C, 700 °C — min	*t*_s_ = 21 h, *t*_d_ = 1 h	130 mL g^−1^	Study the effect of oxidizing agents	77
NFG 50 m: HClO_4_ (i): KMnO_4_: NH_4_NO_3_ (ox)	*T*_i_: 30 °C, *T*_d_: 70 °C, 900 °C 5 min	*t*_i_ = 10 min, *t*_d_ = 4 h	480 mL g^−1^	Recommended use in flame retardant	97
NFG: H_2_SO_4_: HNO_3_ (i): KMnO_4_ (ox)	*T*_i_: RT, *T*_d_: 90 °C, 800 °C 10 s	*t*_i_ = 30 min, *t*_d_ —	Pore volume 0.0185 cm^3^ g^−1^ nm^−1^	Super-capacitors	79
NFG 9 m: H_2_SO_4_ (i): KMnO_4_ (ox)	*T*_i_: RT, *T*_d_: 70 °C, 1000 °C 70 s	*t*_i_ = 1 h, *t*_d_ —	100 times	Wound dressing	116
NGF (500 μm): HClO_4_ (i & ox)	*T*_i_: RT, 1000 °C 60 s	*t*_i_ = 10 s	375 mL g^−1^	Adsorption	60
NFG: HClO_4_ (i): KH_2_PO_4_: KMnO_4_ (ox)	*T*_i_: 30 °C, *T*_d_: 65 °C, 950 °C 15 s	*t*_i_ = 50 min, *t*_d_ = 24 h	420 mL g^−1^, 245 m^2^ g^−1^	Adsorption	17
NGF: H_2_SO_4_: HNO_3_ (i): H_2_O_2_ (ox)	*T*_i_: RT, *T*_d_: —, 1100 °C	*t*_i_ = 1 h, *t*_d_ = —	*ρ* = 12 mg cm^−3^	Adsorption	53
NGF (510 μm): HClO_4_ (i): KMnO_4_: (CH_3_CO)_2_O (ox)	*T*_i_: RT, 1000 °C 50 s	*t*_i_ = 10 s	315 mL g^−1^	Oil sorption	44
NGF: H_2_SO_4_ (i): (NH_4_)_2_S_2_O_8_ (ox)	*T*_i_ = 30 °C, *T*_d_: 105 °C, 500 W 60 s	*t*_i_ = 20 min, *t*_d_ = 24 h	267 mL g^−1^	Recommended use is oil sorption	119
FG 10 m: H_2_SO_4_ (i): K_2_S_2_O_8_ (ox)	*T*_i_ = 50 °C, *T*_d_:—, 800 W 40 s	*t*_i_ = 300 s, *t*_d_ = —	455 mL	Oil sorption	120
NGF 10 m: H_2_SO_4_ (i): H_2_O_2_ (ox)	*T*_i_ = *T*_s_ = −5 °C, *T*_d_: 60 °C, 700 W 50 s	*t*_i_ = 35 min, *t*_s_ = 12 h, *t*_d_ = 12 h	500 mL g^−1^	Oil sorption	9
NGF (500 μm): HClO_4_ (i): Cu (NO_3_)_2_ (ox)	*T*_i_: RT, 800 W 40 s	*t*_i_ = 20 s	484 mL g^−1^, 114.86 m^2^ g^−1^	Adsorption	93
EG-1: NGF: H_2_SO_4_ (i): H_2_O_2_: C_4_H_8_O_2_S: C_5_H_9_NO: KMnO_4_ (ox)	*T*_i_: 30 °C, *T*_d_: 105 °C, 500 W 1 min	*t*_i_ = 95 min, *t*_d_ = 2 h, for each EG	—	Recommended use are graphene preparation and adsorbents	113
EG-2: NGF: H_2_SO_4_ (i): (NH_4_)_2_S_2_O_8_ (ox)					
NGF 100 m: H_2_SO_4_ (i): H_3_PO_4_: KIO_4_ (ox): KMnO_4_ (ox): H_2_O_2_ (ox): HCl	*T*_i_: RT, *T*_d_: 120 °C, 1000 W 2 min	*t*_i_ = 25 h, *t*_d_ = 6 h	16.5 mL g^−1^, 13.06 m^2^ g^−1^	—	54
NGF >1.25 mm: HClO_4_ (i) KMnO_4_: (CH_3_CO)_2_O (ox)	*T*_i_: RT, 360 W 50 s	*t*_i_ = 10 s, 60 s	244 mL g^−1^, 147.5 m^2^ g^−1^	Oil sorption	118
NGF >1.25 mm: HClO_4_ (i) KMnO_4_: (CH_3_CO)_2_O (ox)	*T*_i_: RT, 720 W 40 s	*t*_i_ = 10 s	29.95 m^2^ g^−1^	Adsorption	45
NGF: H_2_SO_4_ (i):H_3_BO_3_: KMnO_4_ (ox)	*T*_i_: RT, 350 W 30 s	*t*_i_ = 3 min	—	Adsorption	51
NGF 1.25 mm: H_2_SO_4_ (i): H_2_O_2_ (ox)	*T*_i_: RT, 720 W 30 s	*t*_i_ = 100 min	196.67 mL g^−1^	—	121
NGF (500 μm):HClO_4_ (i & ox)	*T*_i_: RT, 800 W 60 s	*t*_i_ = 10 s	524 mL g^−1^	Adsorption	60
NGF: H_2_SO_4_ (i): H_3_PO_4_ (i): KMnO_4_ (ox)	*T*_i_: RT, *T*_d_: 80 °C, 350 W 30 s	*t*_i_ = 3 min, *t*_d_ = —	70 mL g^−1^	Adsorption	52
NGF (40 μm): HNO_3_ (i): KMnO_4_ (ox)	*T*_i_: RT, 700 W 60 s	*t*_i_ = 5 min	212.33 mL g^−1^	Recommended use are production of graphene and oil adsorption	117
NGF (510 μm): HClO_4_ (i): KMnO_4_: (CH_3_CO)_2_O (ox)	*T*_i_: RT, 600 W 50 s	*t*_i_ = 10 s	565 mL g^−1^	Oil sorption	44

a NGF: natural graphite flakes, EG: expanded graphite, m: mesh, i: intercalator, ox: oxidant, *T*_i_: intercalating temperature, *T*_s_: standing temperature, *T*_d_: drying temperature, RT: room temperature, *t*_i_ = intercalation time, *t*_s_: standing temperature, *t*_d_ = drying time, *t*_e_: expansion time, Trs: thermal shock, IR: irradiation power.

## 4. Role of expanded graphite in oil sorption

The surface area of graphite significantly increases during the expansion process owing to the formation of abundant pores, resulting in favourable adsorption properties. Expanded graphite has been widely used in oil spill from water surface.^17,19,44,92,122–124^

**Fig. 13:**
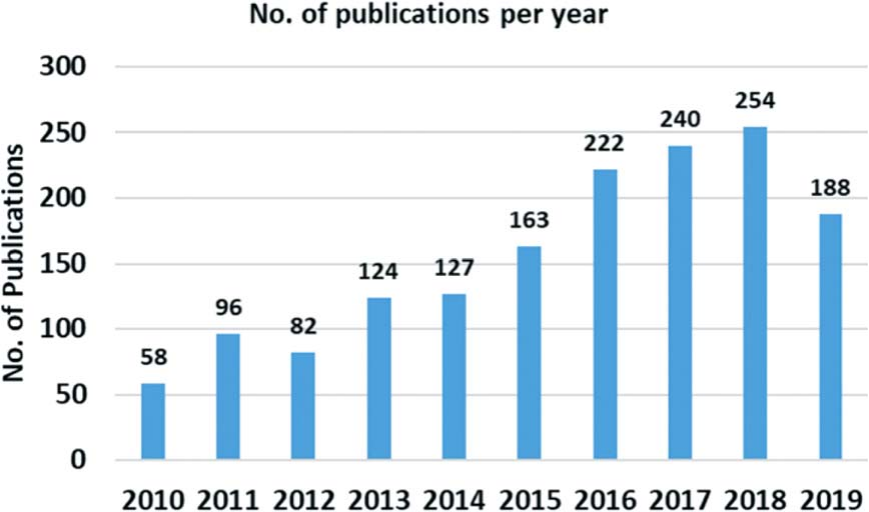
Statistical plot for the number of publications per year on materials for oil recovery applications. The keyword used for the search is “material in oil spill application.” Reprinted with permission from ref ^125^.

Expanded graphite is a highly prospective material due to its unique properties including very low density with typical apparent densities of 0.002–0.01 g cm^−3^, high aspect ratio, high porous structure, closest electrical/thermal conductivity and thermal/chemical stability to natural graphite and ease of preparation and low cost.^72^ Expanded graphite has indeed gained significant attention as a promising material for adsorption applications, particularly in the field of oil sorption. Many interesting articles have been published so far on expanded graphite for oil spill recovery this is evident from the increasing number of publications focused on expanded graphite as an adsorbent between 2010 and 2020, as shown in Fig. 13.^125^

The growing interest in expanded graphite as an adsorbent can be attributed to its unique properties, such as high porosity, large surface area, and excellent adsorption capacity. The expanded structure of graphite provides numerous interconnected pores and channels, enabling efficient adsorption of various substances, including oils and hydrocarbons. Fig. 13 likely depicts a graphical representation or trend analysis showing the rising trend of publications related to the use of expanded graphite as an adsorbent during the specified time period. This indicates the increasing recognition and exploration of expanded graphite's potential in addressing environmental challenges related to oil spills and contamination. The extensive research and development in this area highlight the growing importance of expanded graphite as a versatile and effective adsorbent material with promising applications in oil sorption and environmental remediation.

### 4.1 Potential use of expanded graphite in oil sorption

Inagaki and Toyoda were indeed early pioneers in recognizing the high effectiveness of expanded graphite for oil sorption applications. Their work conducted two decades ago, shed light on the remarkable oil sorption capabilities of expanded graphite. Their research likely demonstrated that expanded graphite exhibits a strong affinity for oils, allowing for efficient sorption of oil from water or when in direct contact with pure oil. This characteristic makes expanded graphite a promising material for oil spill cleanup, environmental remediation, and other oil sorption applications. The ability of expanded graphite to selectively adsorb and retain oils while repelling water is attributed to its unique structure and surface properties. The expanded structure creates a highly porous material with a large surface area, providing ample space for oil molecules to be adsorbed and trapped within its interconnected pores. The early work by Inagaki and Toyoda played a significant role in establishing the foundation for further research and development in utilizing expanded graphite as an effective sorbent material for oil spills and related environmental challenges. Their findings have since paved the way for continued exploration and optimization of expanded graphite-based sorbents for efficient oil sorption and environmental remediation.^126^

In addition, Thinh and his team^40^ prepared magnetic of expanded graphite by coating CoFe_2_O_4_ particles on expanded graphite using the sol–gel method, researchers aimed to develop magnetic expanded graphite with enhanced sorption capacities for oil spill treatment. The results of the study demonstrated successful deposition of CoFe_2_O_4_ particles onto the surface of EG, indicating uniform and effective coating. The as-synthesized magnetic EG-CoFe_2_O_4_ composite exhibited high sorption capacities for different types of oils, namely fuel oil (FO), diesel oil (DO), and crude oil (CO). Among these oils, the highest sorption capacity was observed for fuel oil. This finding suggests that the EG-CoFe_2_O_4_ composite has great potential as a promising material for oil spill treatment, where the sorption of fuel oil is of particular interest. The sorption capacities of the EG-CoFe_2_O_4_ composite were quantified, with the highest sorption capacity observed for fuel oil at 54.13 g g^−1^. Although slightly lower, the composite also exhibited considerable sorption capacities for crude oil (50.79 g g^−1^) and diesel oil (42.12 g g^−1^). These results highlight the potential of the magnetic expanded graphite composite, EG-CoFe_2_O_4_, as an efficient sorbent for the removal of different types of oils from water or other environments. The magnetic properties of the composite, along with its high sorption capacities, make it a promising candidate for oil spill treatment and environmental remediation applications.

In a comparative study conducted by Pham *et al.*,^118^ two different approaches, conventional thermal heating and microwave irradiation methods, were investigated for the fabrication of expanded graphite from locally available flake graphite sources in Vietnam. The objective of the study was to evaluate the effectiveness of these methods in producing expanded graphite for the purification of oil–contaminated water. The results of the study revealed that expanded graphite obtained from both methods exhibited multilevel pore structures, indicating the presence of various pore sizes and interconnected voids. The specific surface area, which is an important parameter influencing adsorption capacity, was measured for the expanded graphite samples. Under optimal processing conditions, the surface area of expanded graphite obtained from the microwave irradiation method was found to be 147.5 m^2^ g^−1^, while the surface area of expanded graphite produced through conventional heating was 100.97 m^2^ g^−1^. Furthermore, the as-synthesized expanded graphite obtained from the microwave irradiation method demonstrated higher adsorption capacities for diesel oil, crude oil, and fuel oil compared to the expanded graphite obtained through the conventional heating method. This suggests that the microwave irradiation method resulted in expanded graphite with improved adsorption performance for hydrocarbon pollutants. The findings of this study highlight the potential of microwave irradiation as an effective technique for the fabrication of expanded graphite with enhanced adsorption properties. The higher surface area and superior adsorption capacities of the microwave-synthesized expanded graphite make it a promising material for the purification of oil–contaminated water, offering potential applications in environmental remediation and oil spill cleanup efforts.

K. H. Wu *et al.*^19^ conducted a study where they synthesized a magnetic expanded graphite (MEG) composite using the explosive combustion method and black powder. The expanded graphite obtained from this process exhibited an expansion volume of 300 mL g^−1^ and a specific surface area (*S*_BET_) of 34.8 m^2^ g^−1^. The researchers investigated the sorption behaviour of the EG/Fe_3_O_4_ composite for the removal of heavy oil, engine oil, and diesel oil from water. The results revealed that the composite exhibited high sorption capacities for these oils. Specifically, the sorption capacity for heavy oil was measured at 87.70 g g^−1^, while engine oil and diesel oil showed sorption capacities of 53.20 g g^−1^ and 44.22 g g^−1^, respectively. These findings indicate that the EG/Fe_3_O_4_ composite possesses excellent sorption properties, making it a promising material for the removal of heavy oil, engine oil, and diesel oil from water. The magnetic properties of the composite, coupled with its high sorption capacities, offer potential applications in oil spill remediation and environmental cleanup processes.

A recent work by Tarango-Rivero *et al.*^9^ proposed a process for the production of expanded graphite that aimed to reduce the consumption of sulfuric acid by utilizing only two intercalation chemicals. They conducted experiments to evaluate the effects of milling, peroxide concentration, and microwave expansion on the process. The results of their study demonstrated that the proposed route led to the production of expanded graphite with high specific volumes and elevated oil adsorption rates. The expanded graphite exhibited a high selectivity for oil–water separation and demonstrated rapid adsorption capabilities. Among the samples tested, the 0–2 and 0–1 samples showed the highest oil adsorption capacities, reaching values of 111.8 ± 5.6 (g g^−1^) and 95.3 ± 3.8 (g g^−1^), respectively. These findings highlight the effectiveness of the proposed process in producing expanded graphite with excellent oil adsorption properties, indicating its potential for applications in oil spill cleanup and water purification.

**Fig. 14:**
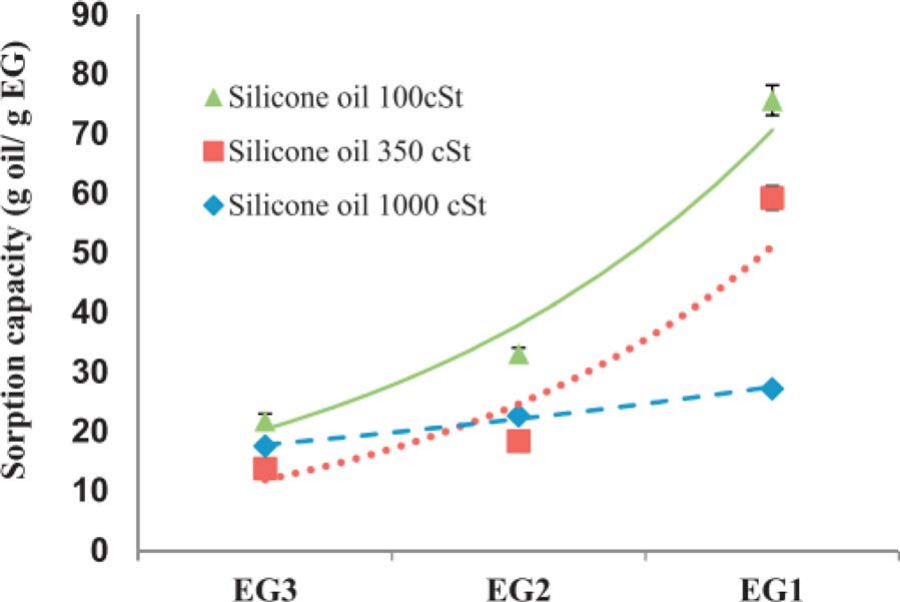
The oil sorption capacities of EGs prepared at different expansion temperatures. Reprinted with permission from ref. 72.

Indeed, the expansion temperature plays a crucial role in determining the oil sorption capacities of expanded graphite. According to Goudarzi and Hashemi^72^ higher expansion temperatures lead to increased sorption capacity due to the higher porosity achieved during the expansion process. When graphite is subjected to high temperatures, the intercalation agents used to expand the graphite, such as sulfuric acid or other oxidants, generate gases that cause the interlayer spacing of the graphite to increase. This expansion results in the formation of a highly porous structure with interconnected voids and channels. The porosity of the expanded graphite is directly related to its ability to sorb oil. Larger pores and voids provide more space for oil molecules to penetrate and be trapped within the structure. Consequently, EG specimens prepared at higher temperatures tend to have a higher porosity, allowing for greater oil sorption capacity. On the other hand, if the pore size distribution of the EG specimens is too small, large oil molecules may face difficulty diffusing into these restricted pores. Therefore, it is desirable to have a suitable range of pore sizes in the expanded graphite structure to facilitate the effective sorption of oil molecules of varying sizes. By optimizing the expansion temperature, researchers can control the porosity and pore size distribution of the expanded graphite, tailoring its sorption capacity for specific applications. This knowledge helps in designing EG materials with enhanced oil sorption capabilities, making them efficient and effective for oil spill cleanup and other oil sorption scenarios (Fig. 14 and Table 3).

**Table 3:** Comparison of adsorption capacity of EGs^a^

EG adsorbent type	Adsorbent material	*S*_BET_ (m^2^ g^−1^) or density g cm^−3^	Maximum sorption capacity (g g^−1^)	Recycle number	Ref.
EG	Heavy oil & crude oil	0.006 g cm^−3^	86 & 76	5	126
EG	Engine oil & kerosene	0.0051 g cm^−3^	56 & 32	—	24
MEG	Fuel oil, diesel oil, and crude oil	—	54.13–50.79–42.12	4	40
EG *via* microwave irradiation	Diesel oil, crude oil, and fuel oil	147.5	74–50–44	—	118
MEG	Heavy oil, engine oil and diesel oil	34.8	87.73–55.69–53.06	3	19
50% H_2_O_2_ 0–2 EG & 30% H_2_O_2_ 0–1 EG	Diesel fuel	Interlayer spacing 0.382 nm	0–2 EG 111.8 ± 5.6 & 0–1 EG 95.3 ± 3.8	6	9
EG1, EG2, EG3, EG4, and EG5	Silicone oil (viscosity of 100 cSt)	31, 28, 25, 27, 13, and 5	29.8, 15.1, 11.1, 23.2, 9.6, and 13.2	—	72
EG blocks	Diesel oil	0.008–0.024 g/cc	46	—	110
EG block	Diesel, kerosene, and engine oil	—	47, 49, and 63	6	25
Mesoporous graphite	Engine oil (viscosity 90 cP)	100.12	85	—	92
EG	Kerosene oil and engine oil	125	40 &125	5	60
EG	Lubricating oil	476	—	8	53
EG	Crude oil, diesel oil, and gasoline	245	123.3, 76.5, and 61.4	3	17
EG	Engine oil, kerosene oil, and hydraulic oil	52.78	102, 94, and 87	—	44
EG-3	Pump oil & vegetable oil	30.26	235 & 86	5	120

a MEG: magnetic expanded graphite.

### 4.2 Nature of expanded graphite adsorption mechanism

For an adsorbent to be considered good, it should have a large sorption capacity, perform rapidly, and exhibit high selectivity. Expanded graphite has a combination of physical–chemical properties, including high porosity, hydrophobicity–oleophilic balance, low density, and chemical stability, which makes it a valuable material for oil removal applications. It offers efficient oil sorption, easy collection, and potential reusability, contributing to environmental remediation efforts.^17,42,53,72^

The highly hydrophobic nature of EG allows it to selectively absorb oil and repel water, thereby improving the effectiveness of separating oil and water. When expanded graphite is arranged in a layered structure, it exhibits a significant presence of conjugated delocalized π-electrons on its surface, attributed to the sp^2^ hybridization of carbon atoms. These π-electrons attract other π and σ electrons, resulting in the formation of π–π coupling. In the case of aromatic compounds, there is the formation of π–π electron coupling or stacking interaction. In hydrocarbons like engine oil, the σ electrons within the absorbed molecules can couple with the π-electrons on EG, thereby enhancing the oil absorption capability.^91,93,126^

**Fig. 15:**
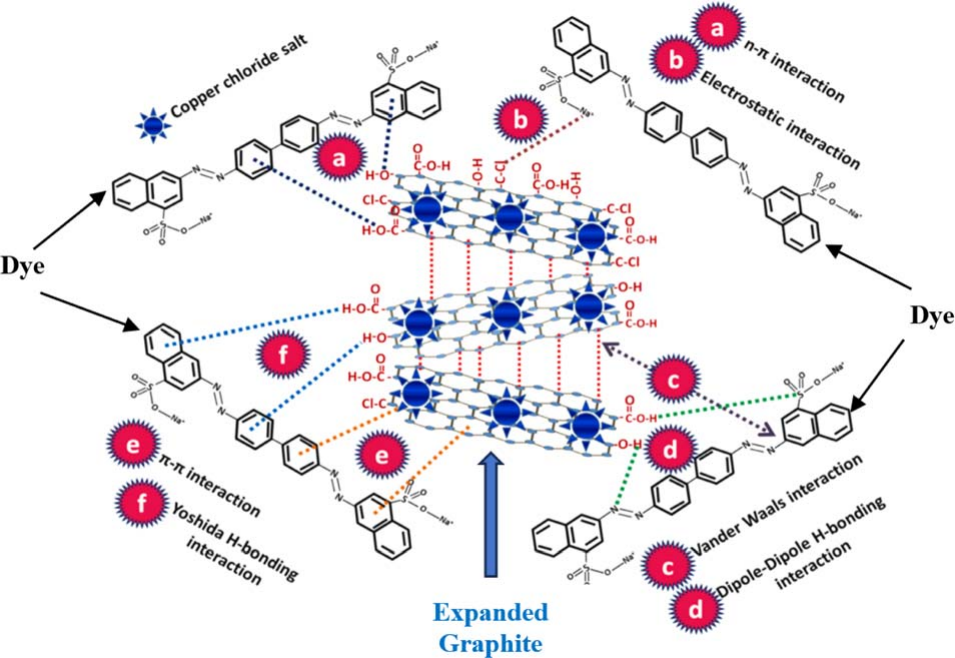
Potential interactions that contribute to the process of dye adsorption on the EG. Reprinted with permission from ref. 93.

In the π–π interaction, the oxygen-containing functional groups on the EG surfaces function as electron donors, while the oil acts as an electron acceptor. Surface functional groups on EG, such as –COOH and –OH, play specific roles in the adsorption of oil. The π–π interaction mechanism is based on the concept of charge transfer occurring between the π electrons of the oil and the π electron distribution on the EG surface. This charge transfer enhances the oil adsorption capacity of the adsorbent. Notably, the EG molecule features two polar nitro-functional groups, known for their strong electron-accepting properties, resulting in a robust π–π interaction between EG and the oil (Fig. 15).

### 4.3 Recyclability

The main drawback associated with the adsorption technique is the cost of the adsorbent materials, which elevates the overall treatment process cost. Thus, recycling and reusing the adsorbent makes it affordable, promotes sustainable development, and allows for green chemistry-based engineering processes. Not only must EG remove oil from water efficiently, but both the oil and EG must be recovered and recycled properly; nevertheless, the porous structure of EG is readily damaged during the regeneration process.^41,110,127^ While dedicated recycling techniques for expanded graphite in adsorption are still under development, there are promising approaches based on thermal methods, solvent extraction, or combinations thereof. Further research is crucial to optimize effectiveness, cost, and environmental impact for a more sustainable life cycle of this valuable material. Different techniques have suggested for separating oil from saturated EG; physical process including filtration,^126^ manual squeezing,^94^ heating^9^ which are practical methods. One of the drawbacks of using the physical method is that the amount of oil recovered decreases significantly as the recycling times increase, due to the oil being trapped in the pores or on the surface of EG. Additionally, the structure and appearance of the particles can be altered, and there are energy costs associated with the process. Another practical separation method is chemical extraction, different solvents have been proposed to separate the oil from the saturated EG, hexane,^18^ petroleum ether,^19^ ethanol.^32^

## 5. Conclusions and outlook

This section highlights the source, structure, synthesize, marketing and application of graphite and expanded graphite. Graphite the raw materials for the expanded graphite which consist of two types, natural graphite and synthesize graphite. Among various carbon-based materials, expanded graphite has garnered significant interest. However, its production and regeneration processes require further development. Expanded graphite has gained global attention due to its unique properties, including low density, high porosity, and electrical conductivity. These intrinsic properties make it a promising material for a wide range of applications, such as fuel cells, electromagnetic interference shielding, catalysts, vibration damping, supercapacitors, biomedical materials, and as an adsorbent for spilled oil. Researchers have early recognized the high effectiveness of expanded graphite in sorbing oil on water or pure oil. The expansion of graphite occurs when the gas pressure increases to a level that exceeds the van der Waals forces between the interlayers. This results in a significant push and allows the graphite to expand along the *C*-axis. Various techniques for preparing expanded graphite involve thermal, chemical, and electrochemical methods. The chemical reaction method is considered cost-effective and easily scalable among these techniques. However, this method typically relies on the use of highly concentrated mineral acids and strong oxidizing agents for the intercalation of graphite. Improper disposal of these chemicals and their by-products can lead to adverse environmental impacts. Therefore, there is an urgent and critical need to explore alternative approaches for the production of expanded graphite that are environmentally friendly and sustainable by addressing the potential environmental concerns associated with the production and disposal of expanded graphite. Consider the ecological impact and sustainability of using such materials.

One of the challenges for the oil sorption is the lack of standardized assessments methods for evaluating the oil sorption performance of thermally expanded graphite. Another challenge related to the mechanical stability of expanded graphite materials during the oil sorption process, especially in the context of real-world applications. Moreover, the scalability of the production of thermally expanded graphite for large-scale oil spill remediation and absorption applications. The final challenge related to the selectivity and specificity of expanded graphite towards different types of oils and hydrocarbons. Discuss its limitations in differentiating between oil and water in complex environments.

Despite significant progress, many opportunities and challenges remain. The following points may be useful for future research.

(1) Explore the potential for functionalizing expanded graphite to enhance its oil sorption capabilities or to impart selectivity for specific oil types.

(2) Discuss the prospects of combining expanded graphite with other materials, such as polymers or nanoparticles, to create composite materials with improved oil sorption properties.

(3) Consider the development of biodegradable expanded graphite-based materials that can be used for oil sorption without causing environmental harm.

(4) Explore the use of advanced analytical and characterization techniques, such as electron microscopy and spectroscopy, to better understand the microstructure and oil sorption mechanisms of expanded graphite.

(5) Discuss the potential applications of expanded graphite in real-world scenarios, such as oil spill cleanup, wastewater treatment, and the removal of oil pollutants from industrial processes.

(6) Address the prospects for expanded graphite materials to meet regulatory and environmental standards for oil spill cleanup.

(7) Consider how expanded graphite aligns with emerging trends in green and sustainable technologies and how it can contribute to cleaner and more eco-friendly oil sorption methods.

(8) Assess the economic feasibility of using expanded graphite for oil sorption and its competitiveness with other sorbent materials.

## Conflicts of interest

There are no conflicts to declare.

## Supplementary Material
